# Creation of a milk oligosaccharide database, MilkOligoDB, reveals common structural motifs and extensive diversity across mammals

**DOI:** 10.1038/s41598-023-36866-y

**Published:** 2023-06-26

**Authors:** Sierra D. Durham, Zhe Wei, Danielle G. Lemay, Matthew C. Lange, Daniela Barile

**Affiliations:** 1 Department of Food Science and Technology, University of California, Davis, One Shields Ave., Davis, CA 95616 USA; 2 Agricultural Research Service, U.S. Department of Agriculture, Western Human Nutrition Research Center, 430 West Health Sciences Dr., Davis, CA 95616 USA; 3 International Center for Food Ontology Operability Data and Semantics, 216 F Street Ste. 139, Davis, CA 95616 USA; 4 Foods for Health Institute, University of California, Davis, One Shields Ave., Davis, CA 95616 USA

**Keywords:** Carbohydrates, Glycomics

## Abstract

The carbohydrate fraction of most mammalian milks contains a variety of oligosaccharides that encompass a range of structures and monosaccharide compositions. Human milk oligosaccharides have received considerable attention due to their biological roles in neonatal gut microbiota, immunomodulation, and brain development. However, a major challenge in understanding the biology of milk oligosaccharides across other mammals is that reports span more than 5 decades of publications with varying data reporting methods. In the present study, publications on milk oligosaccharide profiles were identified and harmonized into a standardized format to create a comprehensive, machine-readable database of milk oligosaccharides across mammalian species. The resulting database, MilkOligoDB, includes 3193 entries for 783 unique oligosaccharide structures from the milk of 77 different species harvested from 113 publications. Cross-species and cross-publication comparisons of milk oligosaccharide profiles reveal common structural motifs within mammalian orders. Of the species studied, only chimpanzees, bonobos, and Asian elephants share the specific combination of fucosylation, sialylation, and core structures that are characteristic of human milk oligosaccharides. However, agriculturally important species do produce diverse oligosaccharides that may be valuable for human supplementation. Overall, MilkOligoDB facilitates cross-species and cross-publication comparisons of milk oligosaccharide profiles and the generation of new data-driven hypotheses for future research.

## Introduction

Mammals are characterized as homeothermic vertebrates with mammary glands. Beings within the class *Mammalia* can be divided into placental mammals, marsupials and monotremes, based on how their young are gestated and born. Placental mammals belong to the clade *Eutheria* and are characterized by fetuses which remain in the uterus of the mother and are nourished by the placenta until a comparatively late stage of neonatal development. In contrast, marsupial offspring undergo a brief uterine gestation followed by a period of further development in the mother’s pouch, where they begin nursing. Diverging even farther, the young of monotremes are laid in eggs and then undergo further development in their mother’s pouch after hatching. While all types of mammalian mothers produce milk to nourish their young after birth, the composition of this milk varies between species^1,2^.

In addition to protein and lipids, carbohydrates are one of the main components of mammalian milk, with oligosaccharides often featuring as the third or fourth most abundant milk component, depending on the species and lactation time point. Milk oligosaccharides are composed of three to twenty monosaccharides. Constituent monosaccharides may include d-glucose (Glc), d-galactose (Gal), *N*-acetyl-d-glucosamine (GlcNAc), *N*-acetyl-d-galactosamine (GalNAc), l-fucose (Fuc), *N*-acetylneuramic acid (Neu5Ac), or *N*-glycolylneuraminic acid (Neu5Gc). Milk oligosaccharides feature either a lactose or, less commonly, a lactosamine unit at the reducing end, and their structures may be extended through the addition of Gal, GlcNAc or GalNAc monomers. Most milk oligosaccharides composed of four or more monosaccharides are divided into two basic categories based on their core structures as either type I or type II (Supplementary Tables 1 and 3). Type I cores feature the structure of lacto-*N*-tetraose (LNT, Gal(β1-3)GlcNAc(β1-3)Gal(β1-4)Glc), while type II cores feature lacto-*N*-neotetraose (LNnT, Gal(β1-4)GlcNAc(β1-3)Gal(β1-4)Glc). Complex milk oligosaccharides with high degrees of polymerization may feature further branching through the linkage of additional α1,3-, α1,4-, or α1,6-linked Gal, GlcNAc, or GalNAc; however, most of these larger structures can still be categorized as having either a type I or type II core^3^. Both type I and type II core structures may also be decorated with α1,2- or α1,3-linked fucose and/or α2,3- or α2.6-linked Neu5Ac or Neu5Gc. Neu5Ac and Neu5Gc are two forms of sialic acid, and oligosaccharides containing either of these monosaccharides are classified as acidic, while those without any sialic acid are categorized as neutral.

Milk oligosaccharides are of particular interest because, although they are assembled at considerable energetic cost to the mother, they are largely undigested by the neonate. Human milk oligosaccharides have been demonstrated to have prebiotic activity, selectively promoting the growth of beneficial bacteria in the infant gut^4–9^. These probiotics then occupy space on the intestinal epithelium, consume human milk oligosaccharides and produce short chain fatty acids, which lower the pH of the gut, making it difficult for pathogens to colonize the infant gut. In addition, the structural homology of milk oligosaccharides to cell surface glycans of the intestinal epithelium allows them to act as receptor decoys to which pathogens may bind in place of host epithelial cells, resulting in the flushing of pathogens from the gut^10^. Human milk oligosaccharides also have anti-inflammatory and immunomodulatory activities and have been shown to decrease gut permeability associated with obesity^11–15^. In addition, the sialic acid found in milk oligosaccharides has been linked to neonatal brain development and learning^16–18^.

The functions of milk oligosaccharides demonstrated to date are dependent upon their structural motifs. As such, oligosaccharides that share monosaccharide compositions may have distinctly different activities depending on their unique isomer structures and glycosidic linkages. Despite the benefits of human milk oligosaccharides, no equally diverse source of bioactive carbohydrates is currently available outside of human milk. Some infant formulas are beginning to be supplemented with prebiotic oligosaccharides, but in most cases the added compounds are not equivalent to those in human breast milk. Despite their demonstrated prebiotic activity, homooligomers like galactooligosaccharides (GOS) and fructooligosaccharides (FOS) lack the structural complexity and compositional diversity of human milk oligosaccharides^19^. The human milk oligosaccharides commercially produced in quantities sufficient for supplementation to infant formula are relatively small, simple structures as more complex human milk oligosaccharide structures have proven to be difficult and expensive to produce through enzymatic synthesis or in genetically modified microbes^20^.

However, many oligosaccharide structures have been identified in the milk or colostrum of non-human mammals with varying degrees of similarity to human milk oligosaccharides. Some non-human mammalian milks are potential sources of oligosaccharides for commercial isolation for supplementation in human infant formulas and functional foods while others represent possible biomedical models for developing a further understanding of the roles of human milk oligosaccharides. The biological significance of variations in milk oligosaccharide profiles among mammalian species is not yet fully understood.

The main challenge in building further understanding of milk oligosaccharides from the existing literature lies in the scattering of the relevant data across decades of publications in dozens of academic books and journals. Although a few carbohydrate databases exist with summaries of large portion of the available carbohydrate literature^21–24^, they feature predominantly plant-, bacterial-, archaeal-, and fungal-derived oligo- and polysaccharides, and protein- or lipid-bound glycans. In addition, these databases are often limited to data originating from a specific type of analysis due to their limited capacity to address structures with different degrees of compositional data available. Complete, targeted searches of mammal-derived glycans like milk oligosaccharides are not easily accessible with these existing resources. Outside of the currently available carbohydrate databases, any cross-publication analysis is hindered by the vast inconsistencies in how milk oligosaccharides have been historically reported, ranging from figures depicting oligosaccharide structures to tables of monosaccharide constituents to full linkage descriptions in the text. These disparate data reporting methods make it prohibitively difficult to make direct comparisons between oligosaccharide profiles reported using different descriptive methods.

The present study overcomes these challenges through the creation of a new database, MilkOligoDB, which reconciles all existing milk oligosaccharide profiles through the use of a standardized form of representing milk oligosaccharide structures regardless of the degree of structural data available. This database facilitates the comparison of oligosaccharides between individual species and across groups of species. In addition, the database holds the potential to contribute to answering questions about the biological significance of specific oligosaccharide structural variations across mammalian milks. When combined with other biological and chemical knowledge bases, together with queries and visualizations, it will also serve as a generator of hypotheses which can be investigated in future milk oligosaccharide studies.

## Methods

### Literature selection

To enable comparisons between milk oligosaccharide profiles of different species, a database, MilkOligoDB, was constructed (Supplementary Tables 1, 2, and 3; https://github.com/Barile-Lab/MilkOligoDB), containing compilations of the existing published milk oligosaccharide profiles for each species discussed herein. For non-human species any studies reporting milk oligosaccharide structures published in a peer reviewed journal or book between January 1970 and July 2022 were considered for inclusion in the database. Publications were excluded from consideration if they had not undergone peer review, were not full articles (i.e. abstract-only publications), did not report original results (i.e. reviews, meta-analyses, secondary analyses of existing published milk oligosaccharides data), did not describe the method through which oligosaccharide analysis was conducted, did not adequately describe the species from which milk was obtained, or were published prior to January 1970 or after July 2022. Due to the high volume of publications on human milk oligosaccharides, a few well-established reviews written by leaders in the field within the most recent decade, and any other study meeting the above criteria that reported 30 or more human milk oligosaccharide structures were used to compile a comprehensive list of human milk oligosaccharides. The number of subjects, milk sample collection method, lactation time point at milk collection, and pooling of milk samples were not used as selection criteria. In cases where the milk oligosaccharides of a species were reported in numerous publications meeting the specified criteria, such as with cow milk, papers were selected so as to build an oligosaccharide profile covering the full scope of identified milk oligosaccharides for the species with minimal redundancy. 113 publications covering the milk oligosaccharide profiles of 77 species were included in the database. MilkOligoDB (https://github.com/Barile-Lab/MilkOligoDB, Supplementary Table 2) also includes information about the metadata for each article, with 3193 entries describing the genus and species, donor, sample, and analysis information, and identifying information for the corresponding publication for each milk oligosaccharide entry.

### Database construction

Oligosaccharide isomers were distinguished in the database based on the compositional information available in the corresponding literature, with varying degrees of identification based on the analytical technique applied in the study. When available, the sequence of monosaccharides, branching, and monosaccharide linkages were specified in the isomer designation. While this strategy allows for the greatest extent of comparison between milk oligosaccharide profiles presented in different studies, there are likely some remaining isomer redundancies. In total, entries for 783 oligosaccharide isomers were included in MilkOligoDB (Supplementary Table 1).

All oligosaccharides are represented by a unique six-part alphanumeric code, comprising five integers separated by underscores, which represent the numbers of hexose, *N*-acetylhexosamine, fucose, *N*-acetylneuraminic acid, and *N*-glycolylneuraminic acid monomers ({Hex}_{HexNAc}_{Fuc}_{Neu5Ac}_{Neu5Gc}) followed by one or more letters designating the isomer. For example, 4_2_1_1_0c is composed of 4 hexoses, 2 *N*-acetylhexosamines, 1 fucose, 1 *N*-acetylneuraminic acid, and no *N*-glycolylneuraminic acid, and has been assigned to the specific oligosaccharide Neu5Ac(α2-3)Gal(β1-3)GlcNAc(β1-3)[Gal(β1-4)[Fuc(α1-3)]GlcNAc(β1-6)]Gal(β1-4)Glc. For publications in which oligosaccharide linkage information was not available, the occurrence of multiple isomers with the same monosaccharide composition (differentiated in the original analysis by retention time, fragmentation pattern, etc.) is noted through the assignment of multiple alphanumeric codes corresponding to the same structural composition. For example, 1_1_0_1_0c and 1_1_0_1_0d, which both correspond to oligosaccharides with a structure of Hex + HexNAc + Neu5Ac, indicate two different isomers of 1_1_0_1_0 with unknown linkages. The full list of oligosaccharide isomers and their respective alphanumeric codes is provided in Supplementary Table 1. Summary statistics for the total number of milk oligosaccharides with specific structural features for each species and their overlap with human milk oligosaccharides are presented in Supplementary Table 3.

### Database queries, visualization, and analysis

The database was queried to compare oligosaccharide profiles for a variety of groups of species, and the ensuing data was transformed into concept maps using CmapTools^25^ to visualize the results.

**Figure 1:**
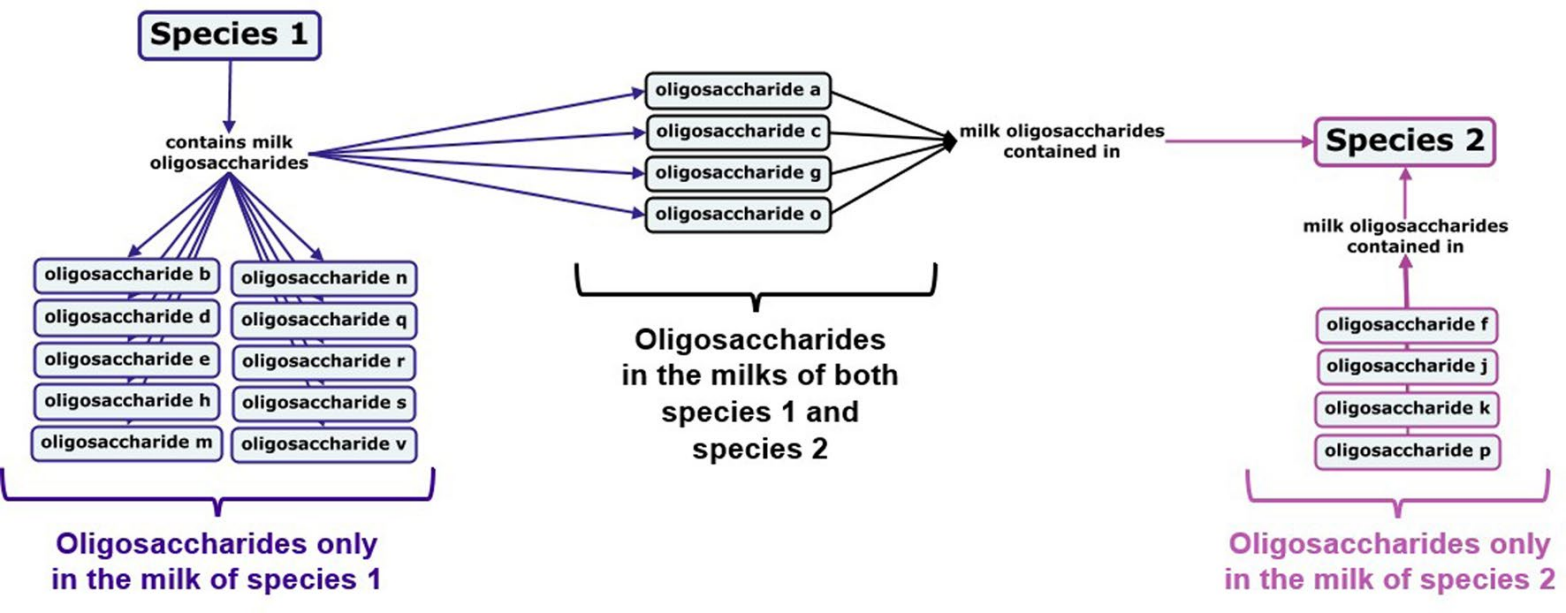
Sample concept map depicting the shared and unique oligosaccharides for two species.

The resulting concept maps can be read from left to right by following the arrows connecting the species names, linking phrases, and oligosaccharides, as exemplified in Fig. 1. Oligosaccharides color-coded as black, with arrows connecting them to multiple species have been reported in the milk of each species to which they share a connecting arrow. Oligosaccharides that are unique to the milk of a single species in a given concept map are color-coded to match that species and bear only a single connecting arrow.

## Results

### Milk oligosaccharides of placental mammals

#### Humans

**Figure 2:**
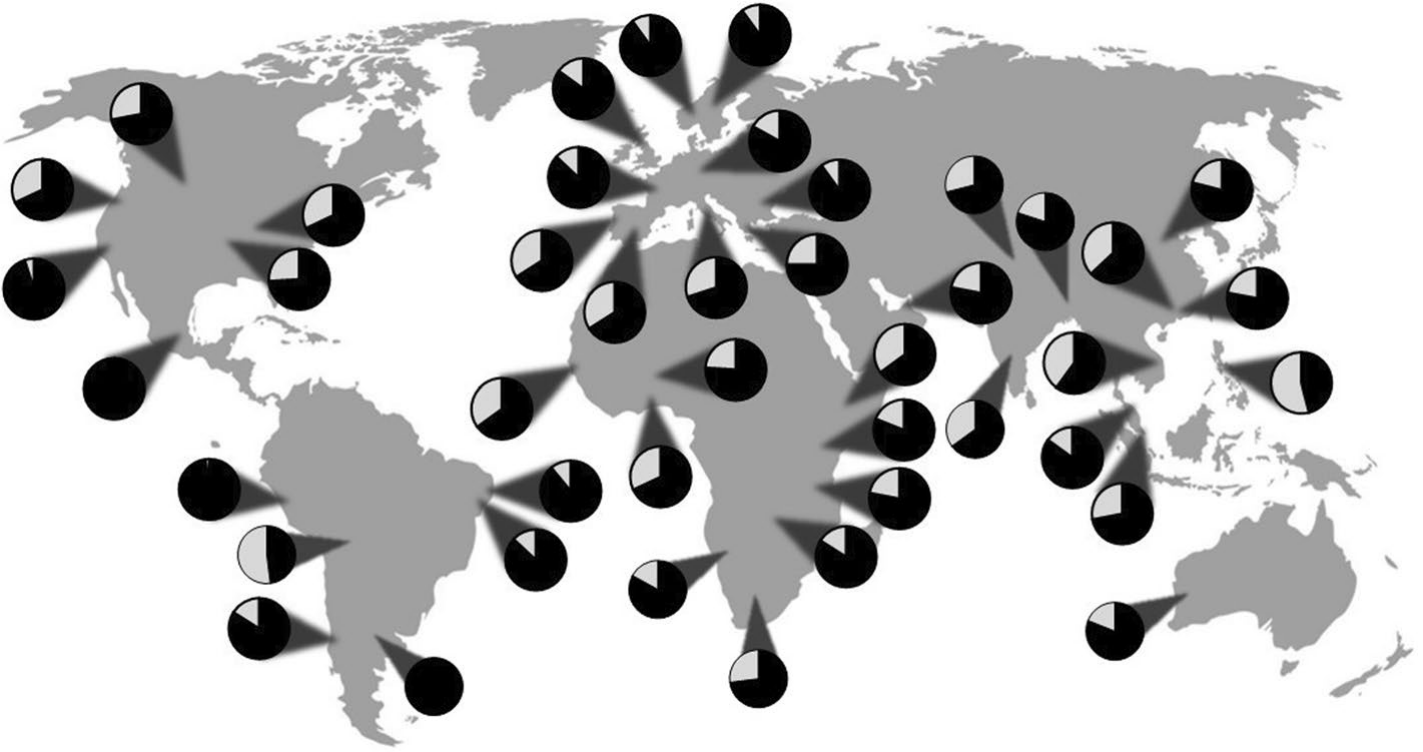
Distribution of secretor status in lactating humans around the world based on the abundance of α1-2-linked fucose in breast milk, where the black sections of the pie charts represent the percent of secretors in the population and the light grey represents the percentage of non-secretors.

Human milk oligosaccharides are by far the most studied set of milk oligosaccharides of any mammalian species. They feature five constituent monosaccharides: Glc, Gal, Fuc, GlcNAc and Neu5Ac, with twelve possible linkages^20^. To date, more than 300 HMO structures have been identified through the use of various analytical techniques, of which 224 unique human milk oligosaccharide structures have been fully elucidated^20,26–30^. Variation in human milk oligosaccharide profiles and concentrations due to the secretor and Lewis status of the lactating person is well documented^31–39^. The secretor gene codes for α1-2-fucosyltransferase, FUT2, and the Lewis gene codes for the α1-3/4-fucosyltransferase, FUT3. Individuals who are secretors express the FUT2 gene and produce milk containing an abundance of α1-2-linked fucose moieties such as 2′-fucosyllactose (2′-FL, Fuc(α1-2)Gal(β1-4)Glc) and lacto-*N*-fucopentaose I (LNFP I, Fuc(α1-2)Gal(β1-3)GlcNAc(β1-3)Gal(β1-4)Glc), while non-secretor mothers produce little to no α1-2-linked fucose-containing human milk oligosaccharides. Individuals who are Lewis positive express the FUT3 gene and produce milk containing oligosaccharides with α1-3- and α1-4-linked fucoses^40^, including 3-fucosyllactose (3-FL, Fuc(α1-3)Gal(β1-4)Glc) and lacto-*N*-fucopentaose II (LNFP II, Gal(β1-3)[Fuc(α1-4)]GlcNAc(β1-3)Gal(β1-4)Glc). The milk of Lewis negative mothers does not contain α1-4-linked fucose moieties but may have human milk oligosaccharides with α1-3-linked fucose units due to the activity of a secretor- and Lewis-independent fucosyltransferase^33^. Secretor status is known to vary between regional, racial, or ethnic groups, as shown in Fig. 2, which contributes to variations in human milk oligosaccharide profiles between cohorts around the world^31,33,37,38,41–54^.

In addition, human milk oligosaccharide concentrations have been shown to vary over the course of lactation, with typical oligosaccharide concentrations in human colostrum as high as 20 g/L but falling to as low as 5 g/L in mature milk^35,36,38,55,56^. The concentration of lactose in human milk is comparatively steady across lactation, at around 60 g/L^57^, resulting in an approximately 1 to 3 ratio of oligosaccharides to lactose in human colostrum and around a 1 to 12 ratio in mature milk. The oligosaccharide profile of human milk is unique in that it does not contain the Neu5Gc form of sialic acid and contains almost no structures with α1-3-linked galactose. Both Neu5Gc and α1-3-linked galactose may be recognized as allergens by humans^58,59^. For most lactating individuals, neutral fucosylated milk oligosaccharides predominate. In addition, the majority of human milk oligosaccharides contain type I core structures. Of the 224 human milk oligosaccharides with full linkage information available, 59.8% have type I cores while only 29.5% have type II cores. (Supplementary Table 3) Although type I oligosaccharides are present in the milk of some other mammals, type II cores prevail in the milk oligosaccharides of most non-human mammals. This combination of structural features—high levels of fucosylation, essentially no Neu5Gc, and mostly type I cores—is uniquely characteristic of human milk oligosaccharides, and rarely found all together in the milks of other mammals. These structural differences, both between donors and between species, are of particular interest because of the strong ties between structure and bioactivities for milk oligosaccharides.

#### Non-human primates

**Figure 3:**
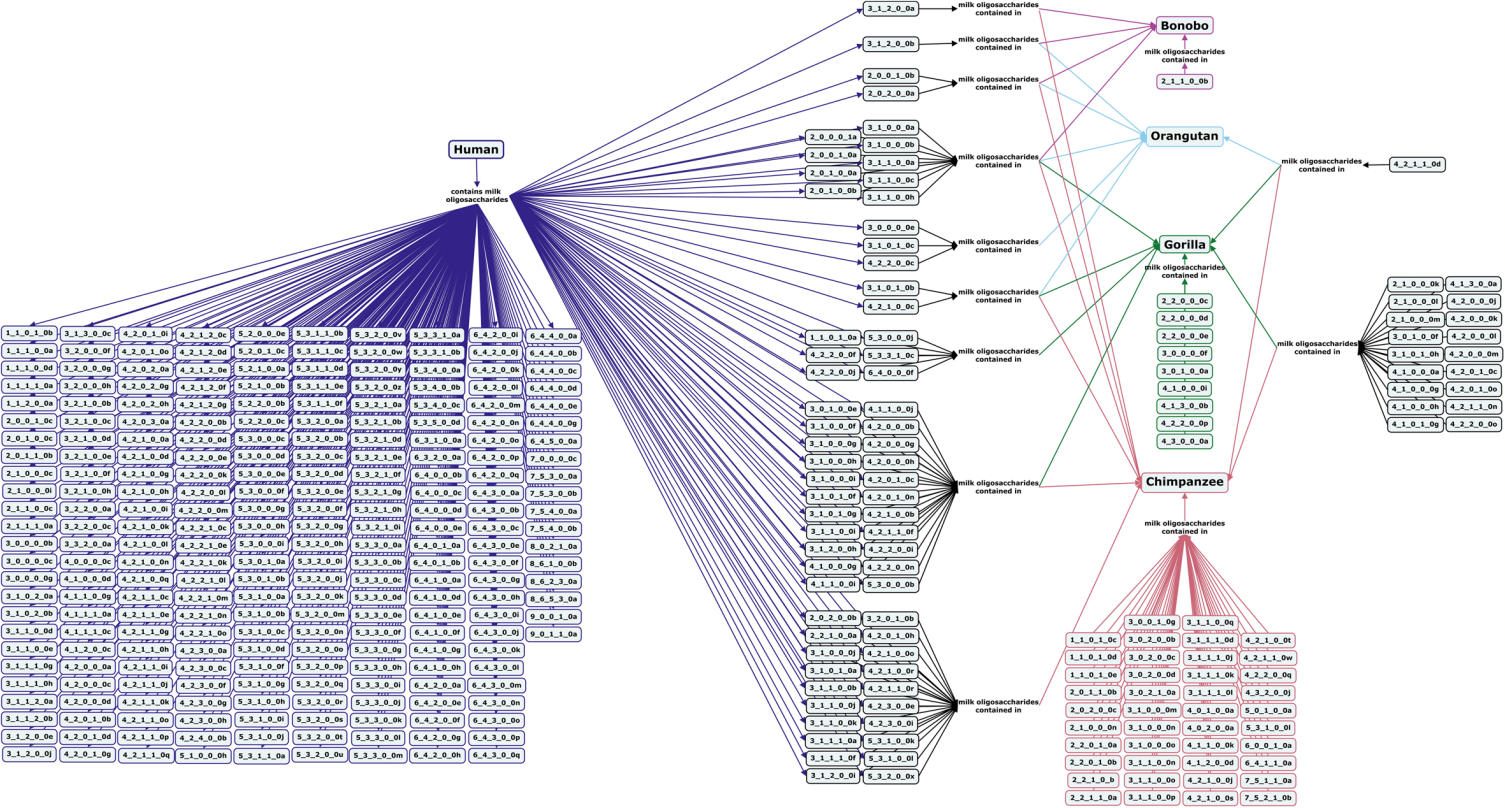
Concept map comparing the milk oligosaccharide profiles of four great ape species, bonobos, orangutans, gorillas, and chimpanzees, with human milk oligosaccharides. Oligosaccharides are designated as the number of {hexose}_{*N*-acetylhexosamine}_{fucose}_{*N*-acetylneuraminic acid}_{*N*-glycolylneuraminic acid} monomers contained in the structure, followed by a letter, to designate the isomer. The full list of oligosaccharide isomer names and their respective alphanumeric codes is provided in MilkOligoDB (Supplementary Table 1). A searchable version of Fig. 3 is available at this link (https://cmapscloud.ihmc.us/viewer/cmap/1Z3CVQ5PP-1XWQCHC-QZZ).

As the closest relatives to humans, data on milk oligosaccharides of non-human primates can aid the understanding of human milk oligosaccharides and their roles. The milk oligosaccharides of a number of non-human primates have been investigated, including those of apes (*Pongidae* and *Hylobatidae*), old world monkeys (*Cercopithecidae*), new world monkeys (*Cebidae, Callitrichidae,* and *Atelidae*), and strepsirrhine primates. Of the primate groups, the great apes, including chimpanzees, bonobos, gorillas, and orangutans, are the closest phylogenetic relatives to humans. Chimpanzee and bonobo milks have oligosaccharide profiles that are about 50% fucosylated with both type I and II cores and a 1 to 4 or 1 to 5 ratio of oligosaccharides to lactose, making them the closest in terms of free carbohydrate composition to human milk. Unlike human milk, however, chimpanzee and bonobo milk oligosaccharides contain Neu5Gc and have more type II than type I core structures (Fig. 3)^60–62^. 2′-FL has been shown to decrease in concentration in bonobo milk over the course of lactation while 3-FL increases in concentration, a trend also observed in human milk^44,62^. In contrast, only α1-2-linked fucose has been identified in gorilla milk, which also contains oligosaccharides with Neu5Gc monomers and both type I and type II core structures^60,61^. Orangutans have milk with a substantially higher ratio of oligosaccharides to lactose (1 to 0.8) than the other great apes, and their milk oligosaccharide profile contains structures with Neu5Gc and predominantly type II cores (Fig. 3)^61,62^.

The only lesser ape for which milk oligosaccharides have been analyzed is the siamang. Although siamang milk’s 1 to 3 ratio of oligosaccharides to lactose is similar to those of the great apes, siamang milk oligosaccharides are the most sialylated (46% of structures contain Neu5Ac or Neu5Gc) of any primate, with only trace amounts of fucosylation^60,61^.

**Figure 4:**
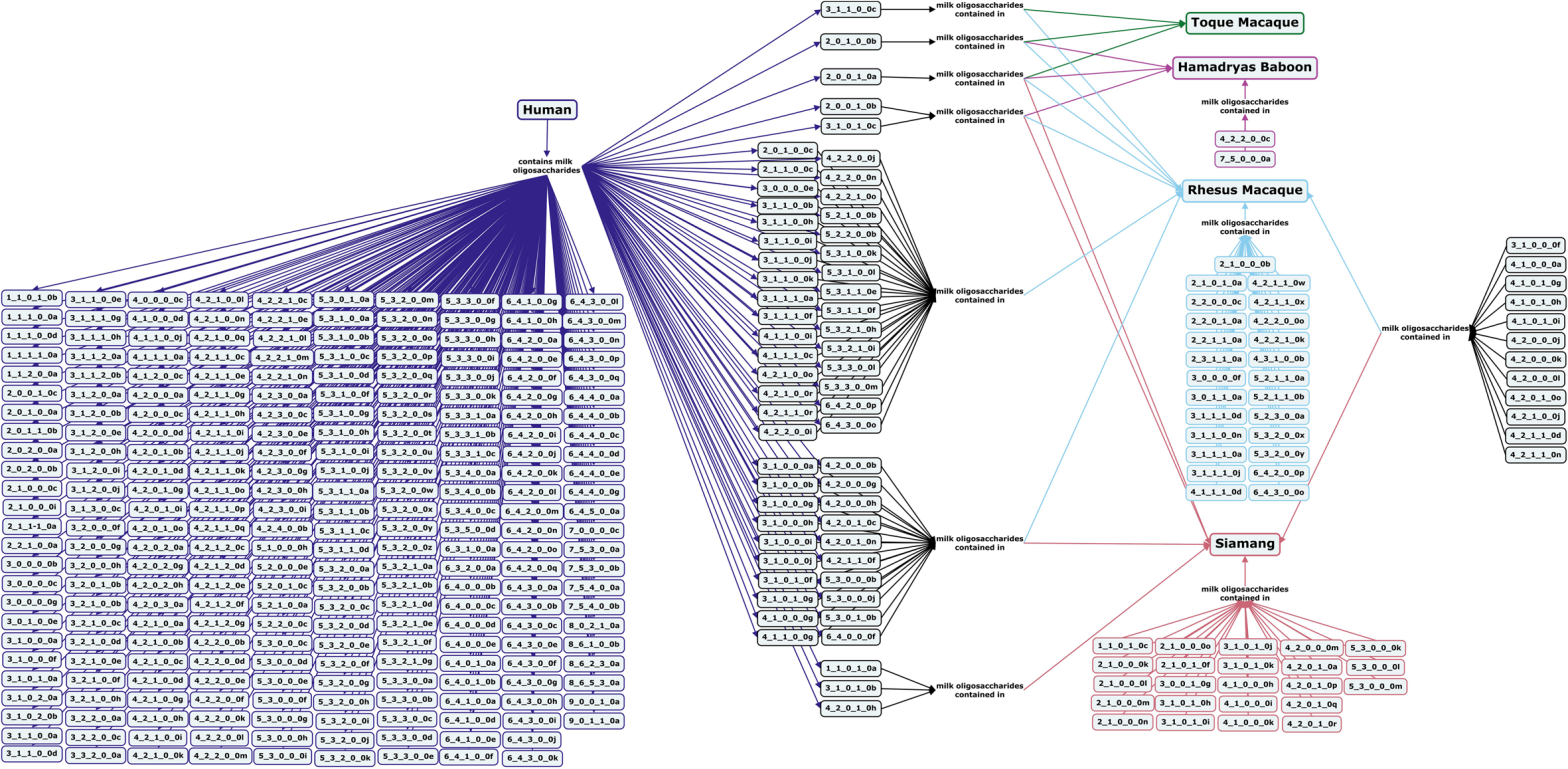
Concept map comparing the milk oligosaccharide profiles of four primate species, toque macaque, Hamadryas baboon, rhesus macaque, and siamang, with human milk oligosaccharides. Oligosaccharides are designated as the number of {hexose}_{*N*-acetylhexosamine}_{fucose}_{*N*-acetylneuraminic acid}_{*N*-glycolylneuraminic acid} monomers contained in the structure, followed by a letter, to designate the isomer. The full list of oligosaccharide isomer names and their respective alphanumeric codes is provided in MilkOligoDB (Supplementary Table 1). A searchable version of Fig. 4 is available at this link (https://cmapscloud.ihmc.us/viewer/cmap/1Z3CW0W43-TJVKQM-W95).

Three species of old world monkeys, hamadryas baboon, toque macaque and rhesus macaque, all have milk oligosaccharides with α1-3-linked fucose moieties, but no α1-2-linked fucose-containing oligosaccharides have been identified^63^. Type I core and Neu5Gc-containing oligosaccharides have both been identified in milk of the rhesus macaque, but not in toque macaque or hamadryas baboon milk (Fig. 4)^60,63^.

**Figure 5:**
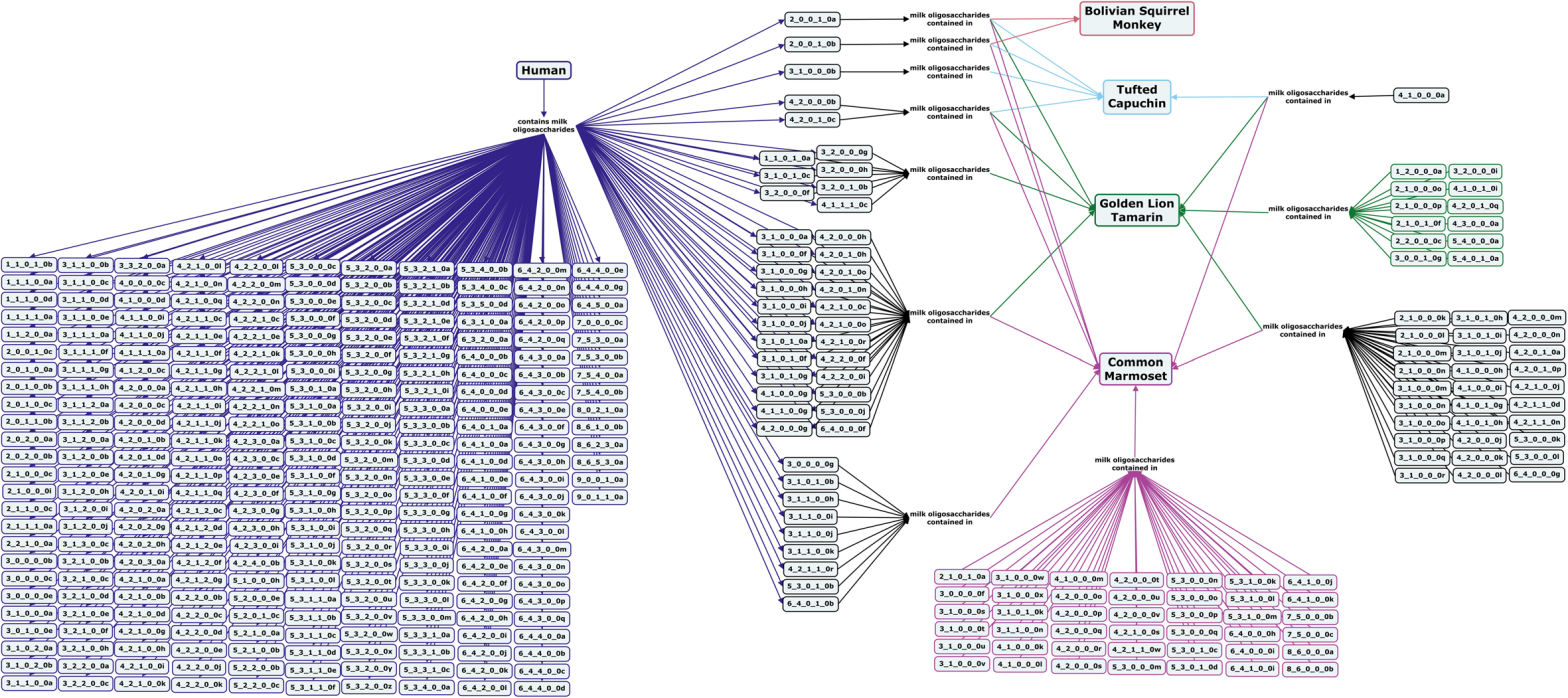
Concept map comparing the milk oligosaccharide profiles of four new world monkey species, Bolivian squirrel monkey, tufted capuchin, golden lion tamarin, and common marmoset, with human milk oligosaccharides. Oligosaccharides are designated as the number of {hexose}_{*N*-acetylhexosamine}_{fucose}_{*N*-acetylneuraminic acid}_{*N*-glycolylneuraminic acid} monomers contained in the structure, followed by a letter, to designate the isomer. The full list of oligosaccharide isomer names and their respective alphanumeric codes is provided in MilkOligoDB (Supplementary Table 1). A searchable version of Fig. 5 is available at this link (https://cmapscloud.ihmc.us/viewer/cmap/1Z3D34NZJ-1XCD8QZ-14DC).

Milk oligosaccharides from three of the five families of new world monkeys have been profiled, including samples of mantled howler, brown capuchin, Bolivian squirrel monkey, golden lion tamarin, and common marmoset milk. With the exception of the common marmoset, for which 21% of identified structures are fucosylated, the milk of new world monkeys appears to contain little to no fucosylated or type I core oligosaccharides (Fig. 5)^60,63,64^.

**Figure 6:**
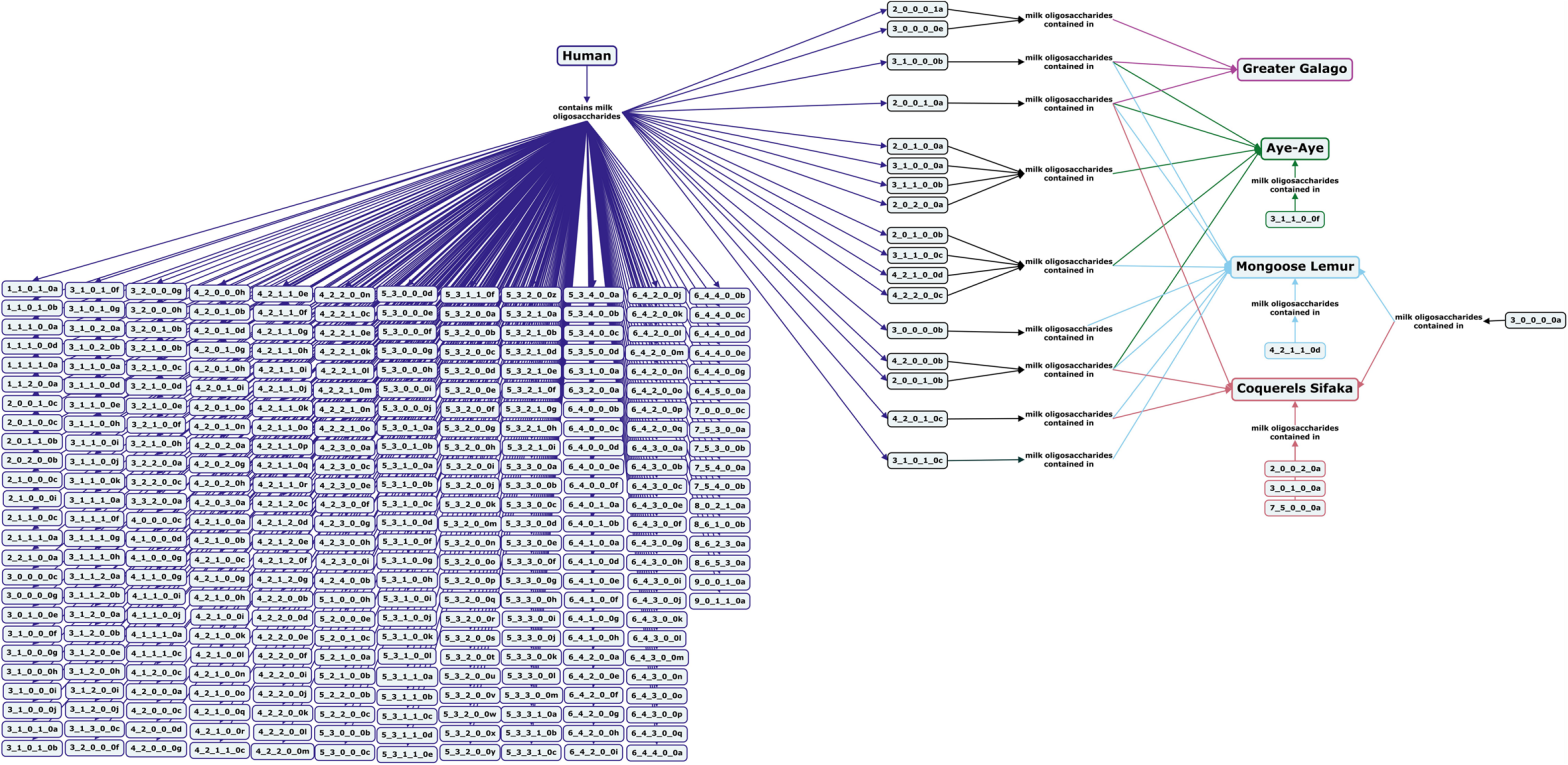
Concept map comparing the milk oligosaccharide profiles of four Strepsirrhine primate species, greater galago, aye-aye, mongoose lemur, and coquerels sifaka, with human milk oligosaccharides. Oligosaccharides are designated as the number of {hexose}_{*N*-acetylhexosamine}_{fucose}_{*N*-acetylneuraminic acid}_{*N*-glycolylneuraminic acid} monomers contained in the structure, followed by a letter, to designate the isomer. The full list of oligosaccharide isomer names and their respective alphanumeric codes is provided in MilkOligoDB (Supplementary Table 1). A searchable version of Fig. 6 is available at this link (https://cmapscloud.ihmc.us/viewer/cmap/1Z3D5M8XM-10RDDWQ-18Z5).

Strepsirrhine primates split off from the lineage of other monkeys and apes an estimated 76–87 million years ago. Milk oligosaccharides from four species in this suborder have been analyzed to date, including the greater galago, aye-aye, mongoose lemur, and Coquerel’s sifaka. The milks of these species have similar ratios of lactose and free oligosaccharides as humans and great apes, but type I core structures have only been identified in aye-aye milk (Fig. 6)^65^.

Overall, primate milk oligosaccharide profiles are more diverse than those of bovine, caprine, or porcine milks and contain similar types of structures as human milk oligosaccharides, but in different proportions (Figs. 3, 4, 5 and 6, Supplementary Table 3)^62^. With an average degree of polymerization (DP) of 4–6, milk oligosaccharide structures of non-human primates tend to be smaller than human milk oligosaccharides (average DP of 7–9)^60^. Current research shows only minimal evidence of correlation between milk oligosaccharide profiles of non-human primates and their phylogenetic relations or social structures^60,65^.

#### Terrestrial carnivores

**Figure 7:**
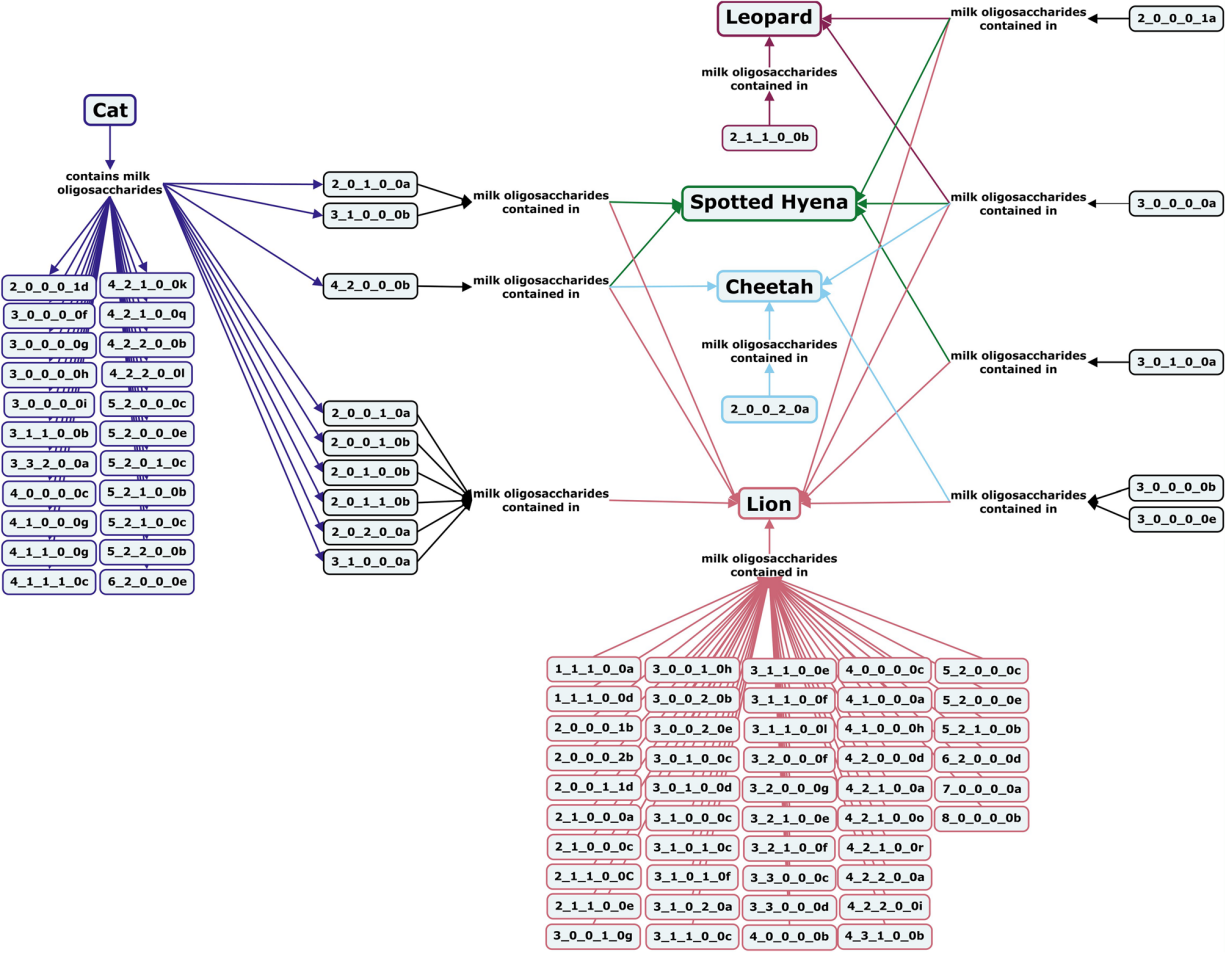
Concept map comparing the milk oligosaccharide profiles of four *Feloidea* species, domestic cats, spotted hyenas, cheetahs, and lions. Oligosaccharides are designated as the number of {hexose}_{*N*-acetylhexosamine}_{fucose}_{*N*-acetylneuraminic acid}_{*N*-glycolylneuraminic acid} monomers contained in the structure, followed by a letter, to designate the isomer. The full list of oligosaccharide isomer names and their respective alphanumeric codes is provided in MilkOligoDB (Supplementary Table 1). A searchable version of Fig. 7 is available at this link (https://cmapscloud.ihmc.us/viewer/cmap/1Z3D5PZXD-2CXHC33-1BLH).

The species within the order *Carnivora* can be divided into two suborders, *Feloidea* and *Canoidea*. A handful of species within *Feloidea* have been the subject of milk oligosaccharide investigations. Primarily small neutral oligosaccharides have been identified in the milk of cheetahs, spotted hyenas, and clouded leopards^66–68^, but larger structures, including a variety of fucosylated oligosaccharides have been identified in the milk of house cats and African lions (Fig. 7)^67,69,70^. Only two acidic oligosaccharides have been identified in *Feloidea* milk, with 6’-SL identified in the milk of house cats and α2-3-Neu5Gc-lactose found in all profiled *Feloidea* milks except cheetah (Fig. 7)^66,67,69,70^. Lions, leopards, and cheetahs all have a milk oligosaccharide to lactose ratio of 1 to 1 or 1 to 2, although lion milk has considerably less lactose (about 27 g/kg) compared to cheetah milk (40.2 g/kg)^67,68,71^.

**Figure 8:**
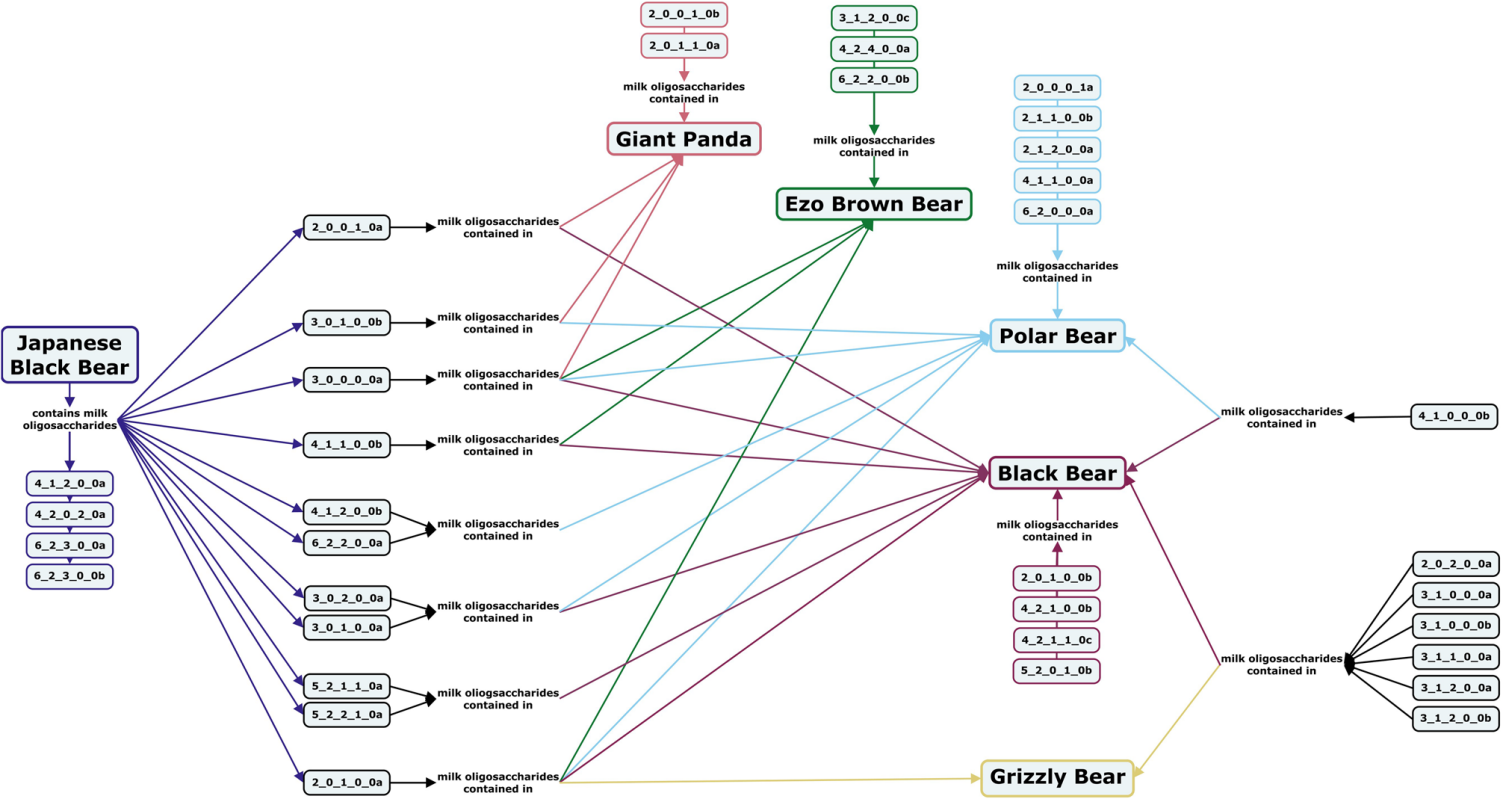
Concept map comparing the milk oligosaccharide profiles of five bear species, including Japanese black bears, giant pandas, ezo brown bears, polar bears, black bears, and grizzly bears. Oligosaccharides are designated as the number of {hexose}_{*N*-acetylhexosamine}_{fucose}_{*N*-acetylneuraminic acid}_{*N*-glycolylneuraminic acid} monomers contained in the structure, followed by a letter, to designate the isomer. The full list of oligosaccharide isomer names and their respective alphanumeric codes is provided in MilkOligoDB (Supplementary Table 1). A searchable version of Fig. 8 is available at this link (https://cmapscloud.ihmc.us/viewer/cmap/1Z3D5X4SB-1MFV5M8-1CGP).

Substantially more investigations into the milk oligosaccharide profiles of species within the *Canoidea* suborder of *Carnivora* have been conducted. The milk oligosaccharide profiles of several species of bears have been studied, including those of the American black bear, Japanese black bear, Ezo brown bear, grizzly bear, polar bear, and giant panda. Both American and Japanese black bear milk contains large α1-2- and α1-3-linked fucosylated oligosaccharides, although only type II core structures were identified in Japanese black bear milk, while both type I and type II core milk oligosaccharides have been identified for the American black bear^62,68,72^. Among the brown bears, milk of the Ezo brown bear is dominated by trisaccharides, especially 2′-FL, while grizzly bear milk contains more DP 4 and 5 fucosylated oligosaccharides with both type I and type II core structures (Fig. 8, Supplementary Table 3)^62,73^. Although the total carbohydrate concentration of polar bear milk remains relatively constant, the oligosaccharide profile varies over the course of lactation, with a high 3′-SL concentration in colostrum but an abundance of isoglobotriose in mid to late lactation milk^74,75^. In contrast, the carbohydrate fraction of giant panda milk increases over the course of lactation, with isoglobotriose (Gal(α1-3)Gal(β1-4)Glc) as the main oligosaccharide throughout^76,77^. Lactose concentrations in bear milk are low at around 1–4 g/kg, which makes them a notable exception to the typically high lactose concentrations in the milk of placental mammals. This low lactose content serves to protect the hibernating mother during lactation both because lipid content is a more efficient method of energy transfer from mother to nursing offspring and because lower lactose concentrations lead to less osmolytic pressure on the milk, lessening the risk of maternal dehydration^68,76,78^.

**Figure 9:**
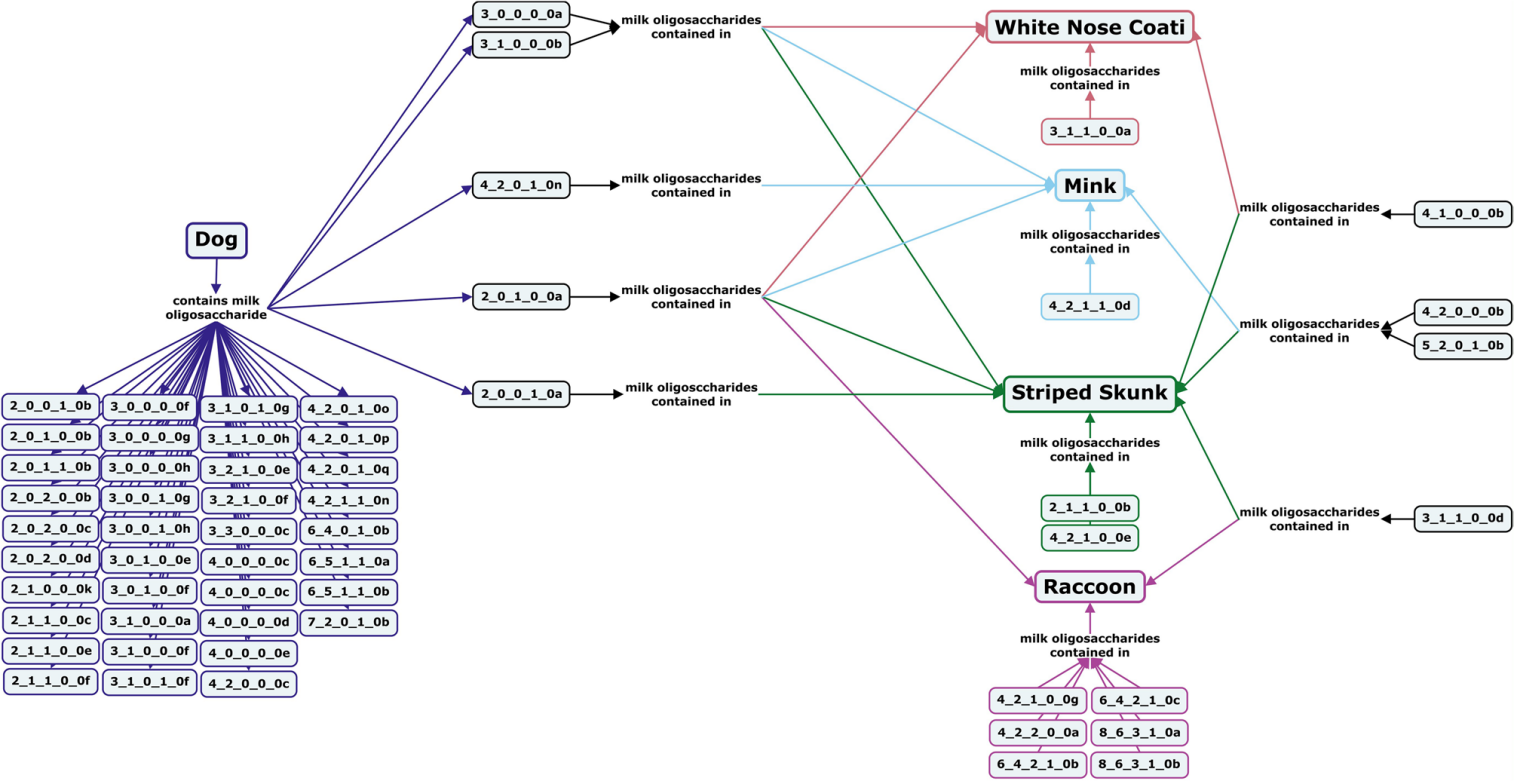
Concept map comparing the milk oligosaccharide profiles of five small *Canoidea* carnivore species, dogs, white nose coatis, minks, striped skunks, and raccoons. Oligosaccharides are designated as the number of {hexose}_{*N*-acetylhexosamine}_{fucose}_{*N*-acetylneuraminic acid}_{*N*-glycolylneuraminic acid} monomers contained in the structure, followed by a letter, to designate the isomer. The full list of oligosaccharide isomer names and their respective alphanumeric codes is provided in MilkOligoDB (Supplementary Table 1). A searchable version of Fig. 9 is available at this link (https://cmapscloud.ihmc.us/viewer/cmap/1Z3D5ZXC0-16VKCMC-1DBG).

Like the larger members of *Canoidea,* milk oligosaccharides are dominated by α1,3-linked galactose-containing cores and Neu5Gc-containing structures are absent from raccoon, striped skunk, mink, dog, and white-nosed coati milk (Fig. 9, Supplementary Table 3)^62,69,79–84^. No acidic oligosaccharides have been reported in mink or white-nosed coati milk, and no type I core structures or α1-3-linked fucose-containing oligosaccharides have been found in the milk of any of the smaller terrestrial carnivores. Unlike most other *Canoidea,* the oligosaccharides identified in raccoon milk include very large structures (DP 13–18) in addition to the smaller neutral fucosylated oligosaccharides (Fig. 9)^79^.

#### Even-toed ungulates

Milks from many species within the *Artiodactyla* order have been analyzed for their oligosaccharide content. These species include ruminants such as cows, goats, sheep, buffalo, antelope, and deer, as well as non-ruminants like pigs.

**Figure 10:**
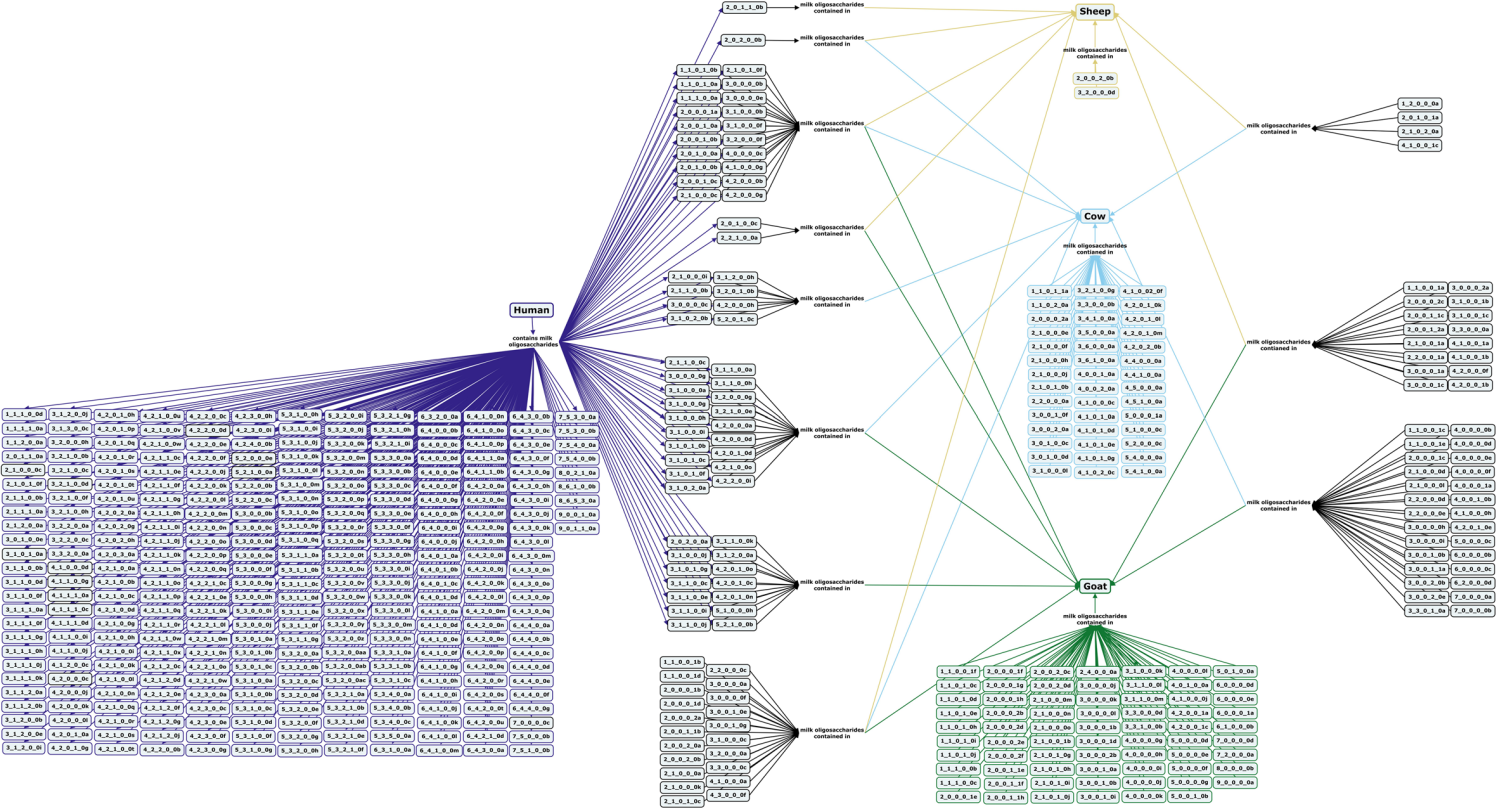
Concept map comparing the milk oligosaccharide profiles of commonly milked Western domesticated mammals including cows, sheep, and goats with human milk oligosaccharides. Oligosaccharides are designated as the number of {hexose}_{*N*-acetylhexosamine}_{fucose}_{*N*-acetylneuraminic acid}_{*N*-glycolylneuraminic acid} monomers contained in the structure, followed by a letter, to designate the isomer. The full list of oligosaccharide isomer names and their respective alphanumeric codes is provided in MilkOligoDB (Supplementary Table 1). A searchable version of Fig. 10 is available at this link (https://cmapscloud.ihmc.us/viewer/cmap/1Z3D65W4C-BF1DY1-1K7P).

Milk and dairy products from cows, goats and sheep are commonly consumed across much of the world. Milk oligosaccharides are present in concentrations of around 1.57 g/L in cow colostrum but fall to between 200 and 300 mg/L in mature cow and goat milk or 2–3 mg/L in mature sheep milk^85–91^. Milk oligosaccharides in these species are much less concentrated than lactose, which is expressed at levels of 49 g/L for cows, 43 g/L for goats, and 48 g/L for sheep^78^. The oligosaccharide profiles for all three species are dominated by acidic structures, with 43% of cow, 46% of goat, and 61% of sheep oligosaccharide structures contain Neu5Ac or Neu5Gc. However, while cow milk features predominantly Neu5Ac-containing oligosaccharide, goat and sheep milk oligosaccharides are largely Neu5Gc-containing compounds (Fig. 10, Supplementary Table 3)^85–88,91–97^. Neutral fucosylated oligosaccharides and type I core structures have been observed in cow and goat milk, but at lower abundances—especially for cow milk—than in the milk of humans and other primates^97–103^. In contrast, most neutral sheep milk oligosaccharides are small, non-fucosylated compounds with no type I core structures reported^86,97,104^. The oligosaccharide profiles of cows and goats have been shown to vary over the course of lactation^93,105,106^ and between animals of different breeds or parities^87,107–109^, in addition to seasonal variation of cow milk oligosaccharides^90,92^. As in humans, genotype may influence the oligosaccharide profiles in goats and cows with changes in goat milk oligosaccharide profiles observed based on the α_s1_-casein production gene *CSN1S1*^110^, and two recent genome-wide association studies strongly correlating changes in milk oligosaccharide expression to several genes in cows^111,112^.

**Figure 11:**
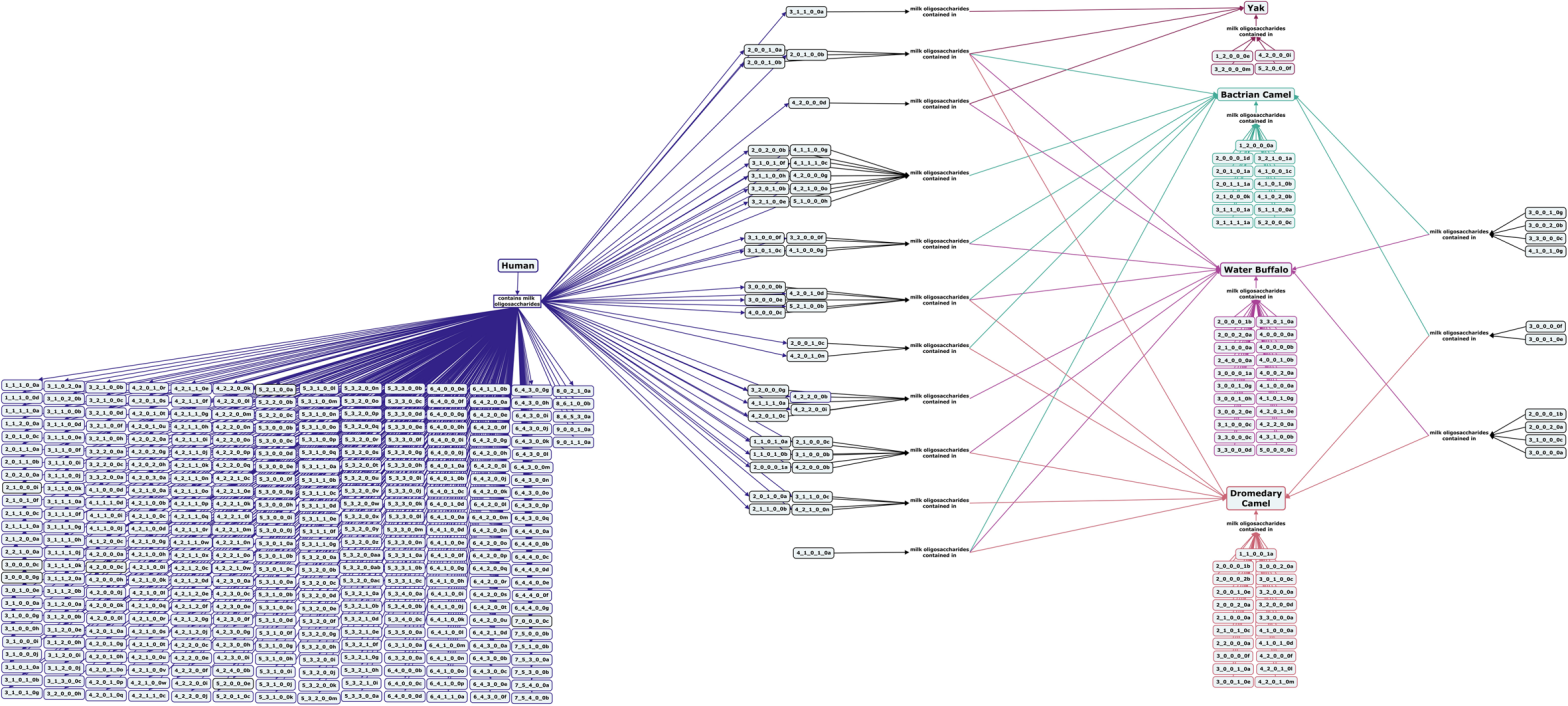
Concept map comparing the milk oligosaccharide profiles of other domesticated mammals milked for human consumption including dromedary and Bactrian camels, water buffalo, and yaks with human milk oligosaccharides. Oligosaccharides are designated as the number of {hexose}_{*N*-acetylhexosamine}_{fucose}_{*N*-acetylneuraminic acid}_{*N*-glycolylneuraminic acid} monomers contained in the structure, followed by a letter, to designate the isomer. The full list of oligosaccharide isomer names and their respective alphanumeric codes is provided in MilkOligoDB (Supplementary Table 1). A searchable version of Fig. 11 is available at this link (https://cmapscloud.ihmc.us/viewer/cmap/1Z3D6JK9T-285NY30-1RMK).

Yak milk is consumed as a food source in regions of China, India, Mongolia, Nepal, and Tibet. Yak milk contains similar levels of lactose and oligosaccharides as dairy cattle^78,113^. Several neutral oligosaccharides have been identified in yak milk, including both an α1,3- and an α1,2-fucosylated structure (Fig. 11)^113–115^. The yak milk oligosaccharide profile also includes 3′-SL and 6’-SL, with substantially more 3′-SL than 6′-SL, similar to the milk of commercial dairy cows^113^.

The oligosaccharide content of buffalo milk has been investigated in several different studies, although not all studies specify what type of buffalo the milk was collected from. The carbohydrate composition of buffalo milk varies significantly between species, with a 1 to 5 ratio of milk oligosaccharides to lactose in water buffalo^116^ but a lactose concentration 500 times higher than the oligosaccharide concentration in African buffalo milk^117^. Overall, it appears that most buffalo varieties likely have predominantly small neutral and acidic oligosaccharide structures, and oligosaccharide profiles that vary over the course of lactation^86,116,118,119^.

Camel milk is frequently consumed in Eastern Europe, north-eastern Africa, and parts of Asia. The majority of camels are dromedary, but Bactrian camels may also be milked as a food source. Compared to other commercially milked mammals, very little research has been done on the oligosaccharide content of camel milk. Dromedary camel milk has low levels of fucose- and Neu5Gc-containing oligosaccharides (14% fucosylated and 12% Neu5GC-containing structures) and no type I cores^97,120^. In both species, acidic oligosaccharides are more abundant than neutral oligosaccharides, but in Bactrian camel milk, fucosylated oligosaccharides outnumber Neu5Gc-containing structures 12 to 7 (Fig. 11) and acidic oligosaccharides decrease in abundance over the course of lactation^120–122^.

Although milk from okapi as well as a number of antelope and deer species has been analyzed, the individual milk oligosaccharides of most non-domesticated species within the *Artiodactyla* order have not been profiled. Oligosaccharides were characterized in Addax milk and found to contain similar concentrations of Neu5Ac and Neu5Gc, with more α2-3-linked than α2-6-linked sialic acid^123^. Seven small neutral oligosaccharides, including 5 fucosylated structures, have been identified in giraffe milk, with only one type II core structure reported^62,116^. Four neutral and acidic oligosaccharides have been identified in reindeer milk too, which was found to be unique in both its lack of Neu5Gc- and α2-6-linked Neu5Ac-containing oligosaccharides and the predominance of phosphorylated oligosaccharides over α2-3-linked Neu5Ac-containing structures^124^. The milk of antelope species contains about 40–50 g/kg lactose, while deer milk has lower lactose concentrations of around 26–28 g/kg^78^. Many deer and antelope milk samples were collected after hunting-related deaths of the animals, but the effects of post-mortem milk sampling on oligosaccharide concentrations is unknown.

The milk oligosaccharide profiles of several porcine breeds have been analyzed, and while minimal variation has been reported between breeds, differences have been observed between pigs of different parities, as with cows and goats^125^. Porcine milk contains very low levels of NeuGc-containing oligosaccharides (less than 2% of structures), making it more similar to human milk than other domesticated large mammals^97,126,127^. Unlike human milk oligosaccharides however, porcine milk oligosaccharides are primarily acidic, with 3′-SL as the most abundant oligosaccharides, and only 16% of pig milk oligosaccharide abundance is composed of fucosylated structures^126,128,129^.

#### Odd-toed ungulates

**Figure 12:**
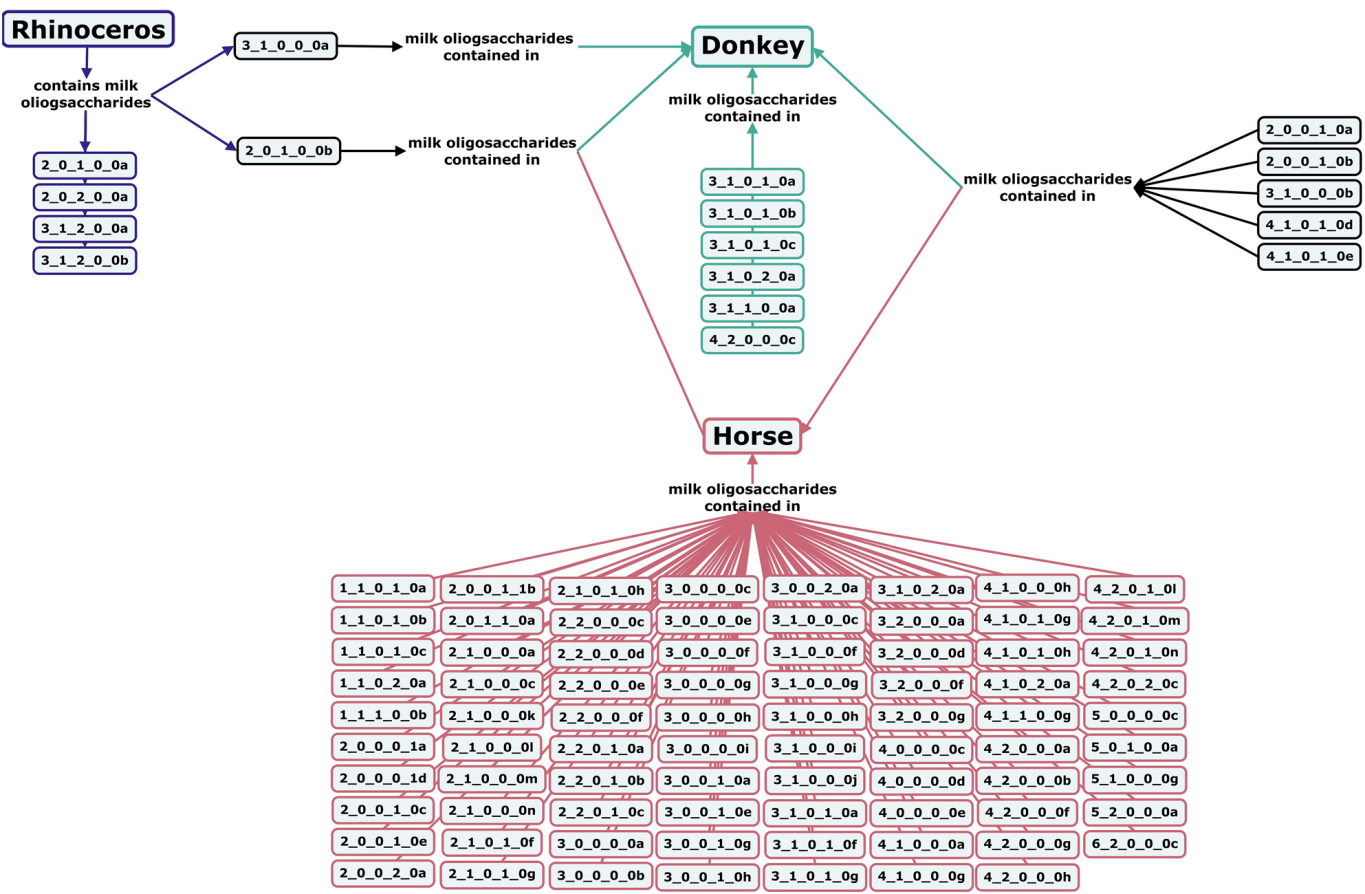
Concept map comparing the milk oligosaccharide profiles of three *Perissodactyla* species, black rhinoceroses, horses, and donkeys. Oligosaccharides are designated as the number of {hexose}_{*N*-acetylhexosamine}_{fucose}_{*N*-acetylneuraminic acid}_{*N*-glycolylneuraminic acid} monomers contained in the structure, followed by a letter, to designate the isomer. The full list of oligosaccharide isomer names and their respective alphanumeric codes is provided in MilkOligoDB (Supplementary Table 1). A searchable version of Fig. 12 is available at this link (https://cmapscloud.ihmc.us/viewer/cmap/1Z3D6SR3X-28601XM-1TLS).

Within the order *Perissodactyla,* only black rhinoceros, donkey and horse milks have been analyzed for their oligosaccharide profiles. Black rhinoceros milk oligosaccharides are predominantly small, neutral fucosylated structures with both α1-2- and α1-3-linked fucose moieties (Fig. 12)^62^. Donkey milk oligosaccharides are primarily small, with 76.2% of identified structures being acidic, of which, 69.2% containing Neu5Ac and 7% containing Neu5Gc structures^130–132^. In horses, the typical milk oligosaccharide concentration in colostrum is 0.217–4.63 g/L but falls to 0.0798 g/L in mature milk, with variation in oligosaccharide profiles between breeds and over the course of lactation^133,134^. The majority of horse milk oligosaccharides are small neutral or acidic structures, with lower levels of Neu5Gc-containing compounds (3.5%) and lactose than cows or goats^97,131–137^.

#### Other terrestrial placental mammals

From the order *Proboscidea*, both Asian and African elephants have undergone milk oligosaccharide analysis. The concentration of milk oligosaccharide changes over the course of lactation in both species, decreasing from 53.7 to around 20 g/L from early to middle lactation in Asian elephants and increasing from 8 to 21.5 g/kg from mid to late lactation in African elephants^138–141^. Isoglobotriose was found to be the most abundant oligosaccharide in the milk of both species, although a range of fucosylated and Neu5Ac-sialylated oligosaccharides, as well as structures with type I and II cores, have also been reported in Asian elephant milk^138,139,141^. African elephant milk contains about 5 times more lactose than oligosaccharides, while Asian elephant milk only contains about twice as much lactose as oligosaccharides^138,140^.

In the order *Pilosa,* milk oligosaccharides have only been analyzed for one species, the giant anteater. Giant anteater milk has a 3.4 to 1 ratio of lactose to oligosaccharides. No fucosylated or α2-3-linked Neu5Ac-containing oligosaccharides have been reported in giant anteater milk, but α2-6-sialylated structures were detected^142^.

The only species from the order *Chiroptera* for which milk oligosaccharides have been profiled is the island flying fox, a bat whose milk was found to lack type I core, fucosylated and Neu5Ac-containing oligosaccharides, but which does feature milk oligosaccharides with Neu5Gc and α1-3-linked galactose, making the oligosaccharide profile of island flying fox milk very dissimilar to that of human milk^143^.

#### Aquatic placental mammals

The order *Cetacea* is divided into marine mammals with and without teeth. Of the toothed cetaceans, milk of a beluga whale and bottlenose dolphins have been analyzed. 3′-SL was the only free carbohydrate identified with certainty in beluga milk; however, because the milk sample was collected at one year postpartum, lactose and additional oligosaccharides may be present in earlier lactation milk^144^. Reports on the oligosaccharide profile of bottlenose dolphin milk vary, with some studies reporting no milk oligosaccharides^145^, and others reporting up to 9 g/L of oligosaccharides^146^. In most baleen whales, lactose has been reported as the most abundant free carbohydrate. Only Neu5Ac-containing oligosaccharides were detected in Bryde’s whale and Sei whale milk^147^, whereas fucosylated, non-fucosylated neutral, and Neu5Ac-containing oligosaccharides were detected in Minke whale milk^144^. All baleen whale milk analyzed in these studies was collected in late lactation, and it is unknown if milk collection post-mortem impacted some oligosaccharide profiles^144,147^.

**Figure 13:**
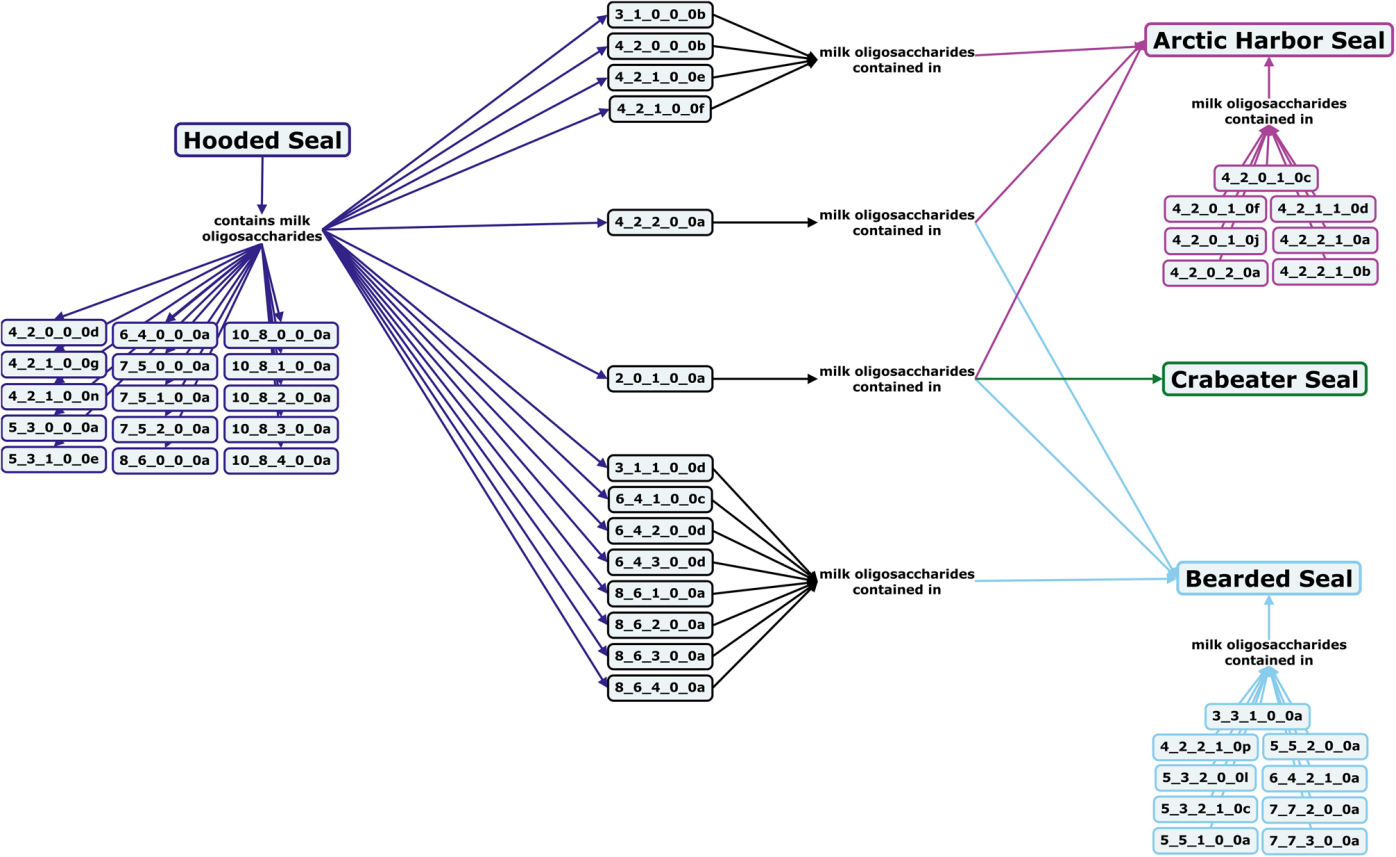
Concept map comparing the milk oligosaccharide profiles of four pinniped species, including hooded seals, arctic harbor seals, crabeater seals, and bearded seals. Oligosaccharides are designated as the number of {hexose}_{*N*-acetylhexosamine}_{fucose}_{*N*-acetylneuraminic acid}_{*N*-glycolylneuraminic acid} monomers contained in the structure, followed by a letter, to designate the isomer. The full list of oligosaccharide isomer names and their respective alphanumeric codes is provided in MilkOligoDB (Supplementary Table 1). A searchable version of Fig. 13 is available at this link (https://cmapscloud.ihmc.us/viewer/cmap/1Z3D6XJ3N-1P0YBBX-1VDJ).

Within the order *Pinnipedia,* no milk oligosaccharides or lactose have been detected in species within the *Otariidae* family, but a number of oligosaccharides have been identified in the milk of *Phocidae* family seals^148,149^ In crabeater seal milk, sialylated and fucosylated oligosaccharides, including 2′-FL have been detected^150,151^. In bearded seal, hooded seal, and arctic harbor seal milk, only type II core structures, α1-2-linked fucosylation, and α2-6-linked Neu5Ac sialylation of oligosaccharides were detected (Fig. 13)^149,152–154^ Milk composition in Weddell seals has been shown to vary over the course of lactation, especially around 2 weeks postpartum when the mothers stop fasting and the total carbohydrate concentration of their milk drops. In early lactation, the carbohydrate fraction of Weddell seal milk is around 90% free oligosaccharides, which is substantially higher than that of terrestrial carnivores. Similar to bears, the low lactose concentration in pinniped milk is likely the result of evolutionary pressure toward rapid nutrient transfer from mother to offspring to more quickly prepare the pup for cold ocean temperatures and increase the size of offspring to hinder predators, a feat more easily achieved through high milk fat rather than lactose content.

The only species for which milk oligosaccharides have been analyzed in the order *Sirenia* is the Florida manatee, whose milk contains little to no lactose and low concentrations of oligosaccharides, consistent with the milk compositions of other aquatic mammals. The 3 milk oligosaccharides that have been identified in Florida manatee milk are neutral structures containing *N*-acetylglucosamine or fucose residues^62,145^.

### Milk oligosaccharides of marsupials

**Figure 14:**
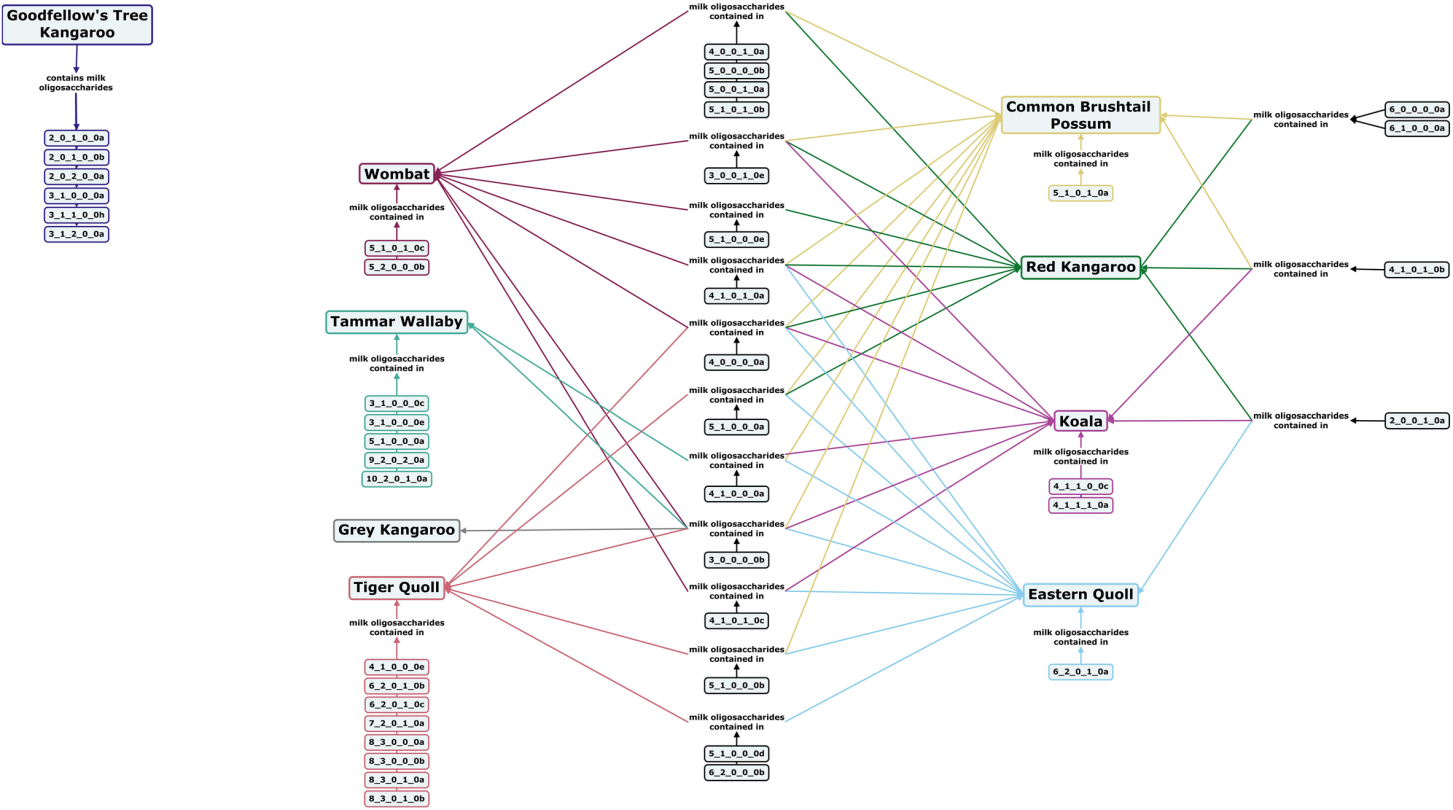
Concept map comparing the milk oligosaccharide profiles of nine marsupial species, including Goodfellow’s tree kangaroo, red kangaroo, grey kangaroo, tammar wallaby, common brushtail possum, wombat, koala, eastern quoll, and tiger quoll. Oligosaccharides are designated as the number of {hexose}_{*N*-acetylhexosamine}_{fucose}_{*N*-acetylneuraminic acid}_{*N*-glycolylneuraminic acid} monomers contained in the structure, followed by a letter, to designate the isomer. The full list of oligosaccharide isomer names and their respective alphanumeric codes is provided in MilkOligoDB (Supplementary Table 1). A searchable version of Fig. 14 is available at this link (https://cmapscloud.ihmc.us/viewer/cmap/1Z3D6ZRX8-2BDKXV4-1W9L).

Unlike most placental mammals, the milk of many marsupials contains little to no lactose, because they lack intestinal brush border lactase, making lactose largely indigestible as a nutrient. In addition, marsupial milk does not contain oligosaccharides with Neu5Gc or type II core structures^155^. Koalas, wombats, and common brushtail possums all have predominantly linear oligosaccharide structures, including acidic milk oligosaccharides, although no α2,6-linked Neu5Ac has been reported in Wombat milk^156–158^. Koalas are one of the only marsupials investigated to date that have milk containing fucosylated oligosaccharides, with 2 out of 10 reported structures containing fucose (Fig. 14)^157^. Interestingly, based on currently available research, Goodfellow’s tree kangaroo milk does not share common oligosaccharide structures with any other marsupial species, and lacks the α1,3-linked galactosyl moiety that is commonly featured in the milk oligosaccharides of other marsupial species^62^. Among other macropods, small and medium neutral non-fucosylated oligosaccharides have been routinely identified, and acidic oligosaccharides in a range of sizes have been reported in red kangaroo and tammar wallaby milk^159–165^. With the exception of the carnivorous tiger quoll and eastern quoll, marsupial oligosaccharides differ from those of most other mammals in that they are primarily small and unbranched structures. (Fig. 14)^166,167^. The carbohydrate content of tammar wallaby, eastern quoll, and common brushtail possum have all been shown to change over the course of lactation, with tammar wallaby milk showing a distinct shift in composition between milk for pouch-bound offspring and more independent, plant-eating joeys that have begun to develop a more ruminant-like digestive system^168–171^. Many marsupial milk oligosaccharide samples were subjected to long-term freezer storage (25–35 years) prior to analysis, but the impact of such storage on milk oligosaccharide profiles is unknown.

### Milk oligosaccharides of monotremes

**Figure 15:**
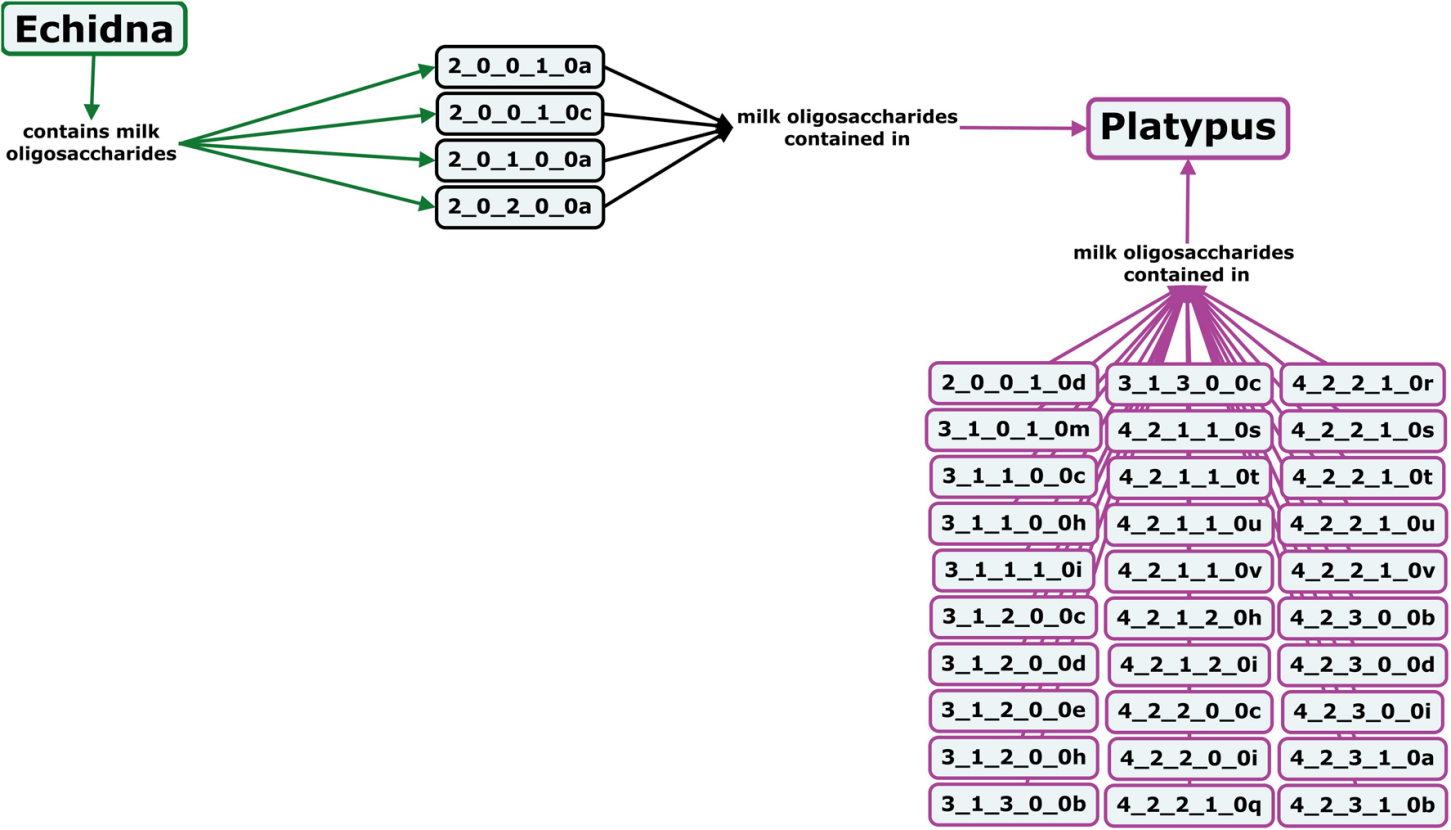
Concept map comparing the milk oligosaccharide profiles of two monotreme species, echidna and platypus. Oligosaccharides are designated as the number of {hexose}_{*N*-acetylhexosamine}_{fucose}_{*N*-acetylneuraminic acid}_{*N*-glycolylneuraminic acid} monomers contained in the structure, followed by a letter, to designate the isomer. The full list of oligosaccharide isomer names and their respective alphanumeric codes is provided in MilkOligoDB (Supplementary Table 1). A searchable version of Fig. 15 is available at this link (https://cmapscloud.ihmc.us/viewer/cmap/1Z4BNCMSS-1RS1P2Q-13BJ).

Monotremes diverged evolutionarily from the ancestors of eutherians and marsupials an estimated 200 million years ago. Although monotremes don’t have nipples, they still secrete milk to nourish their young^172^. Both platypus and echidna milks have levels of sialic acid similar to those of marsupials, but nearly all monotreme milk sialic acid is diacetylated Neu4,5Ac_2_^173,174^. Platypus milk features oligosaccharides with α1,2- and α1,3-linked fucosylation as well as type II core structures, with primarily di- and tri-fucosylated compounds (Fig. 15)^173,175–177^. In contrast, echidna milk oligosaccharides are primarily small, simple, mono-fucosylated or mono-sialylated structures (Fig. 15)^176,178,179^.

## Discussion

### Inter-species milk oligosaccharide comparisons

The unique oligosaccharide profiles of different species are likely the result of evolutionary pressures adapting milk compositions to the needs of both the mother and the neonate^180–182^. Species in which the mothers fast during all or part of lactation appear to produce milk in which oligosaccharides are more concentrated than other carbohydrates including lactose. This pattern has been observed in bears^72–75,183^, *Phocidae* seals^148,149^, and baleen whales^144,147^. In these species, oligosaccharides are likely the main free carbohydrates in milk because energy is transferred from mother to offspring mainly in the form of lipids, not carbohydrates. In some cases, this may be due to the need for rapid offspring growth to increase mobility and avoid predations or the need to increase in neonatal body fat to ensure survival under conditions of extreme cold. In other cases, the lack of mono- and disaccharides in the mother’s milk may instead be the result of evolutionary pressures selecting for the preservation for the mother who, with limited energy stores, must transfer nutrients to her offspring in the manner that results in the least energy and water loss.

In placental mammal species with less-developed neonates at birth, including bears^72,73,76,77,183^, dogs^80^, minks^83^, raccoons^79^, skunks^82^, and primates^60,61,65^ including humans^4,28,48,56,184^ the milk oligosaccharide profiles feature more fucosylated structures than those of species with more precocial offspring. Because the neonates of these species have less-developed immune systems at birth, they are likely more dependent on prebiotic and immunomodulatory compounds, including fucosylated oligosaccharides, delivered by their mother’s milk.

Species with long lactation periods, like elephants and primates including humans, which are phylogenetically distant but developmentally similar in terms of nervous and immune system maturation, show similar trends in oligosaccharide composition over lactation^141^. This may be related to the long, slow growth and extended lactation periods in these species. Although dolphin and toothed whale milk oligosaccharide profiles have not been monitored over the course of lactation, it is possible that similar trends would be observed in these species, given their similarly prolonged lactations.

While some milk oligosaccharides are produced by multiple species, the diversity of oligosaccharides (783 unique isomers in this report) and the many isomers that are unique to a clade or single species is reminiscent of the repertoires of antigen binding molecules in vertebrates. In vertebrates, the adaptive immune system creates antibodies which bind and remove pathogens and these antibodies are highly diverse within and between species due to genetic and structural variation^185^. Milk oligosaccharides have an antibody-like function in that they, too, are able to bind and remove pathogens from the gut. It is likely that the evolutionary pressure of pathogen exposures of different species generated the compositional and structural diversity of milk oligosaccharides.

### Sources of milk oligosaccharide variation within species

In addition to the variation in oligosaccharide profiles that occurs between species, intra-species variations have also been observed. These differences in reported oligosaccharide profiles or concentrations may be due to a number of natural causes. Variation in oligosaccharide profiles between different breeds has been observed in cows^107,109,186^, goats^87^, pigs^125,187^, horses^133^, and dogs^80^. Even within a breed, differences in oligosaccharide abundances have been observed in cows^107^, pigs^125^, goats^87^, and humans^31^ based on parity and, in humans, based on whether a birth is full- or preterm^48,55,188,189^. Genotypes have also been shown to influence oligosaccharide profiles, specifically those associated with α_s1_-casein production in goats^110^ and secretor and Lewis status in humans^31,35,39,47,55,190^. In humans, variations in oligosaccharide profiles have also been associated with the presence of immune diseases, including HIV^45^ and celiac disease^191^. The mother’s diet may also impact the oligosaccharide profile, with a distinct shift observed in Weddell seal milk when mothers stop fasting^148^ and changes observed in the milk of cows fed different diets^192^.

Oligosaccharide profiles are known to vary over the course of lactation too as the needs of the neonate change and they shift away from consuming mother’s milk as their sole food source. Variation in milk oligosaccharides over the course of lactation has been well documented in cows^93,95,105^, pigs^125,126,129,187^, and humans^34–36,44,52,193–197^. Variation in milk oligosaccharide profiles or concentrations of some milk oligosaccharides at multiple lactation points have also been noted in elephants^141^, bonobos^61^, dogs^80^, polar bears^74^, and tammar wallabies^169^. This variation in milk carbohydrate profile of tammar wallaby milk is especially notable because this species can co-express milk of different compositions from different teats simultaneously if nursing both a latched, pouch-bound joey and mobile joey at the same time. With such widespread variation in milk oligosaccharide profiles over the course of lactation, it is exceedingly important that future studies report the lactation time point from which milk is being analyzed. Without this crucial information, studies on the milk oligosaccharides of the same species may seem to present conflicting data, when in fact they may simply be from disparate lactation time points.

### Approximating human milk oligosaccharides: structural and compositional features

**Figure 16:**
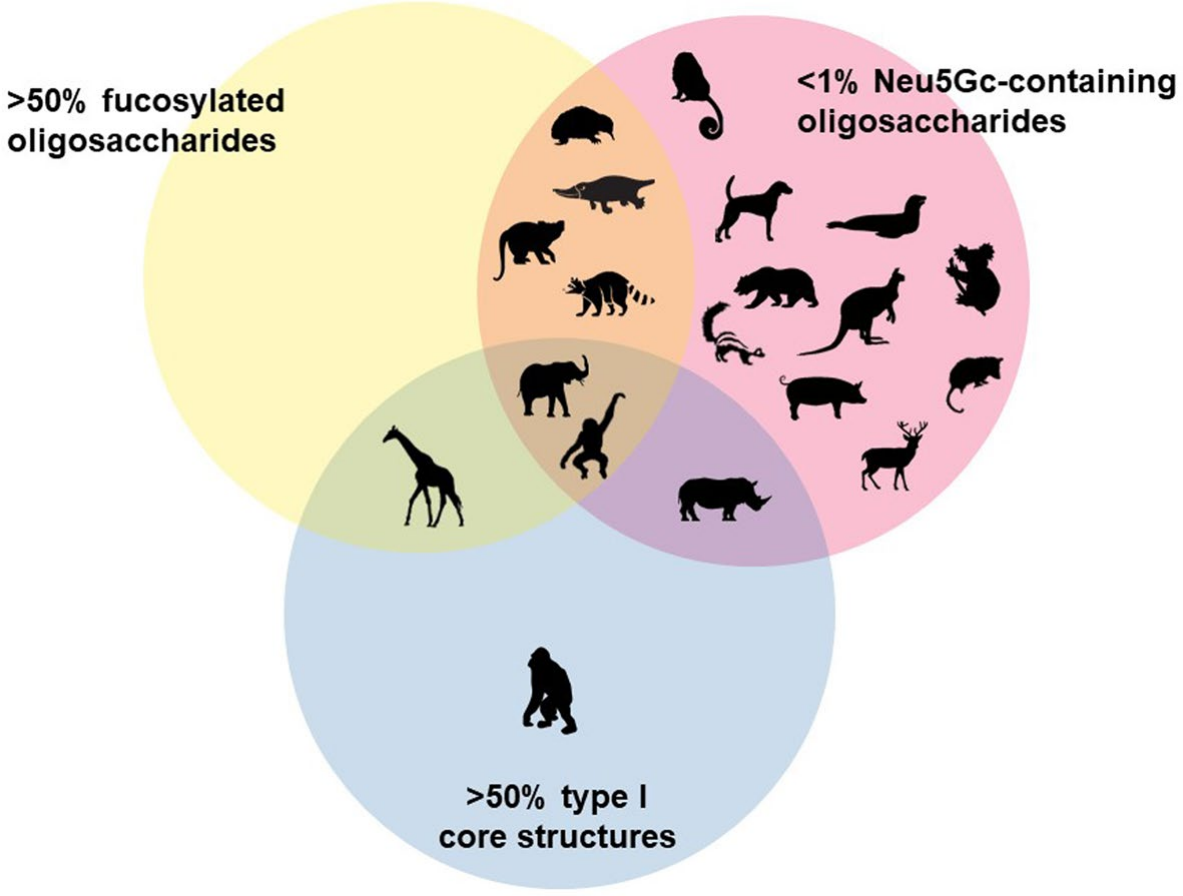
Venn diagram illustrating the overlap between non-human mammals to human milk oligosaccharides, based on the level of fucosylation, abundance of Neu5Gc containing oligosaccharides and the predominance of type I core structures, with comparisons based on the number of oligosaccharide structures identified with the specified characteristics.

Despite the wide sources of variation, several mammalian species produce milk oligosaccharide profiles with characteristics quite similar to human milk oligosaccharides. Marsupials, monotremes, pigs, new world monkeys, *Phocidae* seals, Asian elephants, and terrestrial carnivores express milk with very low levels of Neu5Gc-containing oligosaccharides. Monotreme, elephant, raccoon, skunk, giraffe, chimpanzee, bonobo, and common marmoset milks contain relatively high numbers of fucosylated oligosaccharides. The milks of giraffes, elephants, and most primates have more type I than type II core structures. Other species like lions, cows, horses, and goats may have a greater number of oligosaccharide structures in common with human milk, but these results are skewed by the greater total number of oligosaccharides identified in these species, and it is important to also consider the structural features of the oligosaccharides in these species that are not shared with humans. To most closely mirror human milk oligosaccharides, a milk oligosaccharide profile should have little to no Neu5Gc-containing oligosaccharides, while presenting high levels of a diverse array of fucosylated oligosaccharides and substantially more type I than type II core oligosaccharides (Fig. 16). Based on the currently available research, Asian elephant, chimpanzee, and bonobo milks have the best balance of all three of these features. Summary statistics for oligosaccharide structures and compositions for each species can be found in Supplementary Table 3.

Despite their promising similarities to humans in terms of milk oligosaccharide content, not all of the species listed above are reasonable sources for milk oligosaccharide isolation. Successful milk oligosaccharide isolation at the pilot scale has been demonstrated for both cow and goat milk, and similar techniques could be applied to harness the oligosaccharide available in the milk or dairy streams originating from other commercially milked mammals^86,198–203^. Though not at the same scale as cows, the milk or dairy side streams from producing butter and cheese from horses, Bactrian camels and goat breeds with relatively high concentrations of fucosylated oligosaccharides and low abundances of Neu5Gc-containing oligosaccharides (Figs. 10 and 11), could be used to create supplements for human infant nutrition or for use as a food ingredient in other products for human consumption. In addition, other camelid species like llamas and alpacas, which have the potential to be commercially milked, pose further possibilities for species whose milk oligosaccharide profiles warrant investigation for these purposes. More studies of the milk of these species detailing their full milk oligosaccharide profiles and oligosaccharide concentrations are still needed.

### Database advantages, limitations and future perspectives

The comparisons drawn in this study are inherently limited by the depth and scope of the published studies reviewed herein. In many cases, the published results are only an indication of what the overall profile of the milk oligosaccharides for a given species may look like. A number of studies have been limited by small sample size availability, occasionally with as little as one individual chosen to represent an entire breed or species. Such sweeping assumptions come with the known risk that milk oligosaccharide profiles vary, sometimes widely, between individuals within a group. Factors such as parity, season, location, genotype, captivity status, and days in milk may have inherent influences on milk oligosaccharide profiles. Additional variation in reported results between studies may be due to the application of a wide range of milk collection methods, sample storage conditions and analytical techniques. The work reviewed here spans more than 5 decades, over which time methodology, instrumentation, and commercially available standards for milk oligosaccharide analysis have improved greatly. As the database grows tertiary analysis with additional data collected may yield new insights about the impact of these variables on milk oligosaccharide profiles.

Additionally, because the concentrations of individual oligosaccharides were not reported in most of the reviewed literature, no comparisons of the abundance of particular oligosaccharide classes or structures was made during this analysis. All descriptions of milk oligosaccharide profiles have “more” or “less” of a specific category of oligosaccharides are based on the number of reported structures of that type. As such, the analysis of any milks potentially containing a large number of very low abundant compounds with a given structural feature, or a high concentration of a single oligosaccharide may be skewed by this analysis. Cross-species studies profiling the abundances of a wide range of oligosaccharides are needed to draw more complete milk oligosaccharide comparisons. A study of this nature was undertaken by Albrecht et al. nearly a decade ago^91^, for a limited number of domesticated species, but further investigation is needed, particularly comparing the abundances of oligosaccharides across the species milked for human consumption that have been identified through MilkOligoDB as potential sources of oligosaccharides paralleling those found in human milk.

Further, because milk oligosaccharide entries in the database contain varying levels of detail on the corresponding structures and glycosidic linkages, there may be some inherent redundancies within the compiled oligosaccharide profiles for some species. In particular, this may arise when comparing data from NMR, enzymatic, or standard-based chromatographic isomer identifications that contain complete structural information with less detailed identifications made by mass spectral or chromatographic techniques. Similarly, it is possible that oligosaccharides identified through less detailed techniques for which complete glycosidic linkage data is not available, may appear to be shared between species without actually representing the same oligosaccharide isomer with that composition. For example, an oligosaccharide known to be composed of 3 hexoses may be identified in the milk of both cows and humans, but without further structural detail, we cannot confirm whether these two oligosaccharides share the same isomeric structure. All available structural information was included in each oligosaccharide database entry to make comparisons as accurate as possible, but such comparisons are ultimately limited by the level of structural data available in the original publications.

Despite these limitations, this database and the concept maps derived from it facilitate a cumulative analysis of all existing published milk oligosaccharide profile data that has not been previously undertaken at this magnitude. Reconciling the oligosaccharide data from existing publications into a common format allows for cross-species and cross-publication comparisons that would otherwise be hindered by the unstandardized multitude of textual, tabular, and visual formats in which oligosaccharide profiles are reported. In addition, the concept map format reveals areas that have been comparatively underinvestigated or in which there are substantial gaps or inconsistencies in the existing literature. At its heart, this platform is not only a way to compile data, but also an avenue to generate new data-driven hypotheses for future research. It is not difficult to imagine this data, and its collection going forward, in combination with databases of mammalian offspring gut microbiomes, promoting our understanding of the precise molecular mechanisms by which mammalian milk drives the establishment and maintenance of beneficial gut microbiota, immunomodulation, and brain development. In addition to adding more data, next steps for the MilkOligoDB include development of and linkage to standardized, unambiguous identifiers for all terms, development of a graphical interface that allows users to make and see comparisons across animal milks, research methods, and other experimental details, as well as development of an application programming interface (API), that enables users to query data remotely so that they can include it in their own biological databases of interest.

## Conclusions

All mammals produce milk from mammary glands to suckle their young; however, the OS content of their milk can differ greatly. Although it is unlikely that the milk oligosaccharides of all mammalian species will be profiled in the near future, targeted investigations of the milk oligosaccharides of particular mammals could advance the field on several fronts. Minimal to no research has been done on the milk oligosaccharides of species from nearly half of the 19 orders within the class *Mammalia*. Profiling milk oligosaccharides from species in these relatively untouched orders, including *Dermoptera*, *Insectivora*, and *Lagomorpha* would provide improved understanding of how and why milk oligosaccharides developed from an evolutionary perspective. Further investigation into domestic species that are more commonly milked in non-western countries, such as yaks, camels, water buffalo, llamas, and alpacas would aid in the identification of potential dairy streams from which oligosaccharides could be isolated for supplementation in infant formulas and other nutraceutical products. Additional investigation into the influence of the impact of milk collection conditions, including the impact of oxytocin administration to induce milk let-down, collection of milk post-mortem, long-term storage, and milk oligosaccharide profiles from captive animals versus those in their natural habitats would also provide further context for the interpretation of existing milk oligosaccharide data.

Continued development of the MilkOligoDB and its technical ecosystem of interfaces and update mechanisms will enable new questions to be asked and answered about milk composition across all of mammalia.

## Supplementary Information

Supplementary Tables.

## Data Availability

All data analyzed in this study are present in the Supplementary Materials and available online at https://github.com/Barile-Lab/MilkOligoDB.
